# Exploring the role of gut microbiota modulation in the long-term therapeutic benefits of early MSC transplantation in MRL/*lpr* mice

**DOI:** 10.1186/s11658-025-00716-8

**Published:** 2025-04-18

**Authors:** Quanren Pan, Fengbiao Guo, Jiaxuan Chen, Haimin Huang, Yanyan Huang, Shuzhen Liao, Zengzhi Xiao, Xi Wang, Liuyong You, Lawei Yang, Xuemei Huang, Haiyan Xiao, Hua-Feng Liu, Qingjun Pan

**Affiliations:** 1 Guangdong Provincial Key Laboratory of Autophagy and Major Chronic Noncommunicable Diseases, Department of Nephrology, Affiliated Hospital of Guangdong Medical University, Zhanjiang, China; 2 Department of Clinical Laboratory, State Key Laboratory of Respiratory Disease, The First Affiliated Hospital of Guangzhou Medical University, Guangzhou, China; 3 Department of Anesthesiology, First People’s Hospital of Foshan, Foshan, Guangdong China; 4 Department of Cellular Biology and Anatomy, James and Jean Culver Vision Discovery Institute, Medical College of Georgia, Augusta University, Augusta, GA USA; 5 Clinical Research and Experimental Center, Affiliated Hospital of Guangdong Medical University, Zhanjiang, China

**Keywords:** Mesenchymal stem cells, Systemic lupus erythematosus, Gut microbiota, Tryptophan metabolism, Butanoate metabolism, Aryl hydrocarbon receptor

## Abstract

**Background:**

Systemic lupus erythematosus (SLE), influenced by gut microbiota dysbiosis, is characterized by autoimmune and inflammatory responses. Human umbilical cord-derived mesenchymal stem cell (hUC-MSC) transplantation is an effective and safe treatment for refractory or severe SLE; however, the long-term efficacy and mechanisms of early hUC-MSC therapeutic benefits in SLE need further investigation.

**Methods:**

Here, lupus-prone MRL/MpJ-*Fas*^*lpr*^ (MRL/*lpr*) mice were divided into three groups: the control (Ctrl) group received saline injections, while the MSC and MSC-fecal microbiota transplantation (FMT) groups received early hUC-MSC transplants at weeks 6, 8, and 10. The MSC-FMT group also underwent FMT from the Ctrl group between weeks 9 and 13.

**Results:**

Our results showed that early MSC treatment extended therapeutic effects up to 12 weeks, reducing autoantibodies, proinflammatory cytokines, B cells, and improving lupus nephritis. It also modulated the gut microbiota, increasing the abundance of beneficial bacteria, such as *Lactobacillus johnsonii* and *Romboutsia ilealis*, which led to higher levels of plasma tryptophan and butyrate metabolites. These metabolites activate the aryl hydrocarbon receptor (AHR), upregulate the *Cyp1a1* and *Cyp1b1* gene, enhance the zonula occludens 1 (ZO-1) protein, promote intestinal repair, and mitigate SLE progression. Notably, FMT from lupus mice significantly reversed hUC-MSC benefits, suggesting that the modulation of the gut microbiota plays a crucial role in the therapeutic response observed in MRL/*lpr* mice.

**Conclusions:**

This research innovatively explores the early therapeutic window for MSCs in SLE, highlighting the partial mechanisms through which hUC-MSCs modulate the gut microbiota–tryptophan–AHR axis, thereby ameliorating SLE symptoms.

**Graphical Abstract:**

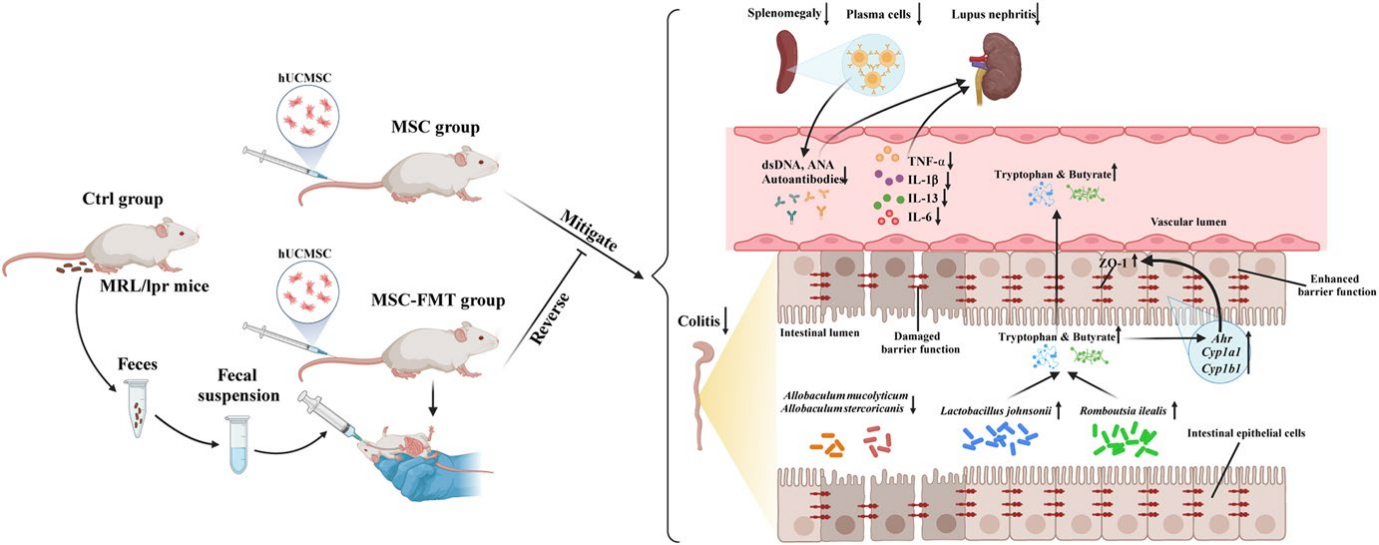

**Supplementary Information:**

The online version contains supplementary material available at 10.1186/s11658-025-00716-8.

## Introduction

Systemic lupus erythematosus (SLE) is a chronic autoimmune disease characterized by excessive B cell activation, autoantibody production, inflammatory cytokines, and multiorgan damage [1]. SLE is a progressive condition, with over 50% of patients developing lupus nephritis (LN), a leading cause of death [2]. The early course of SLE typically shows autoantibody production but may have mild or atypical features not meeting full SLE criteria [3]. Primary treatments for early SLE include antimalarials, statins, and glucocorticoids [4]. Despite these medications’ efficacy, some patients’ conditions remain unmanageable and may progress to end-stage renal disease [5]. Long-term use of these drugs can cause retinal toxicity, infection, and other adverse effects [6].

Gut microbiota dysbiosis significantly contributes to the onset and progression of SLE [7]. In SLE, dysregulation of the gut microbiota can lead to a leaky gut, which is a dysfunction of the intestinal barrier resulting in increased intestinal permeability. This condition facilitates the migration of pathogenic bacteria and toxins to other organs, triggering systemic inflammation [7]. Early intervention targeting the gut microbiota can significantly influence the severity and progression of SLE [8]. Investigating the gut microbiota may provide innovative avenues for early diagnosis, prevention, and therapeutic approaches for SLE.

Mesenchymal stem cells (MSCs) are multipotent cells with strong proliferation and differentiation abilities in vitro. MSCs exhibit low immunogenicity and can regulate the immune system, reducing inflammatory responses [9]. Human umbilical cord-derived MSC (hUC-MSC) transplantation is a highly effective and safe treatment for refractory patients with SLE [10]. MSC transplantation significantly reduced mortality in severe lupus mice, improved renal function, and maintained immune homeostasis [11]. The role of MSCs in modulating gut microbiota has been extensively studied in various pathologies, including inflammatory bowel disease [12], osteoporosis [13], stroke [14], acute lung injury [15], and diabetes [16]. However, whether MSC transplantation can delay SLE progression by ameliorating intestinal dysbiosis and metabolic disorders remains unknown.

In our recent study, we demonstrated that early MSC intervention effectively halts B cell subset differentiation and significantly improves clinical symptoms in MRL/MpJ-*Fas*^*lpr*^ (MRL/*lpr*) mice over a 4-week observation period [17]. Building on this, this study aims to investigate whether early MSC treatment can extend therapeutic benefits to 12 weeks in patients with long-term SLE remission. In addition, it aims to investigate the role of modulating gut microbiota imbalances and metabolic issues (gut microbiota–tryptophan–aryl hydrocarbon receptor (AHR) axis) in hUC-MSCs’ capacity to ameliorate SLE symptoms. Understanding these interactions could lead to novel treatment strategies, offering valuable insights into the gut microbiota’s role in systemic autoimmune responses.

## Material and methods

### Animals

A total of 21 female MRL/*lpr* mice (4-week-old; weight, 20.28 ± 1.4 g) were purchased from Shanghai Slack Laboratory Animal Co., Ltd. (Shanghai, China; license no. SCXK (Hu) 2017-0005) and housed in the SPF Animal Experimental Center of Guangdong Medical University. The mice in this study were housed in standard conditions, including a 12-h light/dark cycle, 22–25 °C temperature, and 40–60% humidity, with unlimited access to food and water. All animal studies complied with the ethical guidelines for researchers by the International Council for Laboratory Animal Science (ICLAS) and were approved by the Guangdong Medical University Animal Care Committee (permission number: GDY2103031; date issued: 25 August 2021). The welfare of the animals was closely monitored both during the application of the experiments and throughout the experiment duration by the employees of the Laboratory Animal Center of Guangdong Medical University.

### Culture and transplantation of hUC-MSCs

hUC-MSCs at passage numbers 5–7 were obtained from Hunan Yuanpin Biotech Co., Ltd. (Changsha, China) and cultured in minimum essential medium alpha basic (cat. no. 12571500, Gibco, USA) with 10% fetal bovine serum (FBS) (cat. no. abs974-500ml, Absin, China) and 1% penicillin/streptomycin (cat. no. 15140122, Gibco, USA) at 5% CO_2_ and 37 °C. The hUC-MSCs exhibited fibroblast-like morphology and plastic adherence, meeting the minimum criteria set by the International Society for Cellular Therapy (ISCT) [18]. Upon reaching 80% confluence, the cells were digested using 0.25% Trypsin–EDTA (1×) (cat. no. 25200056, Gibco, USA), centrifuged at 200*g*, and resuspended in physiological saline. The pH of the physiological saline, used as the carrier for the cells, is strictly controlled between 7.2 and 7.4 to mimic the in vivo environment. To prepare the MSC suspension for injection, we first calculated the average weight of all mice and determined the required cell concentration using the formula 1 × 10^5^ cells/10 g body weight/300 μL. For instance, for mice with an average weight of 30 g, the injection concentration is 1 × 10^6^ cells**/**mL. During the cell collection process, we ensured cell viability and consistency of the injection conditions to meet experimental requirements.

Previous studies defined weeks 8–10 in MRL/*lpr* mice as the early course of SLE [19]. In total, 21 female MRL/*lpr* mice were randomly divided into three groups (*n* = 7 per group): control (Ctrl), MSC transplantation (MSC), and MSC combined with fecal microbiota transplantation (MSC-FMT). Mice in the MSC and MSC-FMT groups received three separate MSC transplants via the tail vein at weeks 6, 8, and 10. Each transplant was administered at these specific time points to ensure consistent and sustained delivery of MSCs throughout the early phase of SLE development. The injection volume for each mouse was calculated on the basis of its weight and the formula (1 × 10^5^ cells/10 g body weight/300 μL). Mice in the Ctrl group received an equal volume of saline (300 μL). Additionally, mice in the MSC-FMT group received FMT from control (Ctrl) group mice of the same age (weeks 9 to 13). FMT was administered via oral gavage every 2 days for a total of 12 sessions. Specifically, fecal samples were collected from Ctrl group mice, and the fecal microbiota suspension was prepared as previously described [20]. The suspension was administered to MSC-FMT group mice at a dose of 0.2 mL per mouse. A detailed description of the FMT procedure is provided in the subsequent section.

### Fecal sample collection and fecal microbiota transplantation

Each mouse was individually placed in a clean, sterilized cage for fecal sample collection. After the mouse defecates, 3 to 5 fecal pellets were collected with sterile tweezers and placed into a 1.5 mL sterile tube. Fecal samples from 15-week-old mice for metagenomic sequencing were immediately placed into liquid nitrogen and then stored at −80 °C for subsequent analysis. To prepare the fecal microbiota suspension, approximately 1 g of fresh fecal pellets was suspended in 5 mL of sterile phosphate-buffered saline (PBS) for mouse fecal microbiota transplantation. The suspension was filtered through gauze to remove large particles, and the filtrate was passed through a 40 μm sterile filter to remove smaller particulate matter. The filtrate was collected in a sterile centrifuge tube and centrifuged at 4 °C, 1000*g* for 10 min to remove insoluble substances and retain the supernatant. Mice in the MSC-FMT group were gavage-fed the fecal microbiota suspension from the same-aged Ctrl group between weeks 9 and 13. The microbial supernatant was transplanted into the mice at a dose of 0.2 mL per mouse, every 2 days, for a total of 12 gavage feedings.

### Euthanasia, sample collection, and processing

Prior to tissue and blood collection, all MRL/*lpr* mice were anesthetized with pentobarbital sodium (100 mg/kg) via intraperitoneal (i.p.) injection. Complete sedation was confirmed by the absence of paw reflex, at which point euthanasia was performed through cervical dislocation. At week 22 postintervention, blood samples were collected via cardiac puncture under terminal anesthesia. Approximately 1–1.2 mL of blood was collected from each mouse, using EDTA K2 anticoagulant tubes to prevent clotting. After collection, the blood was centrifuged at 2000 × *g* for 10 min at 4 °C to separate the plasma from the cellular components. Approximately 0.5–0.6 mL of plasma was obtained per sample, which was immediately aliquoted and stored at −80 °C for subsequent analyses.

**Fig. 1:**
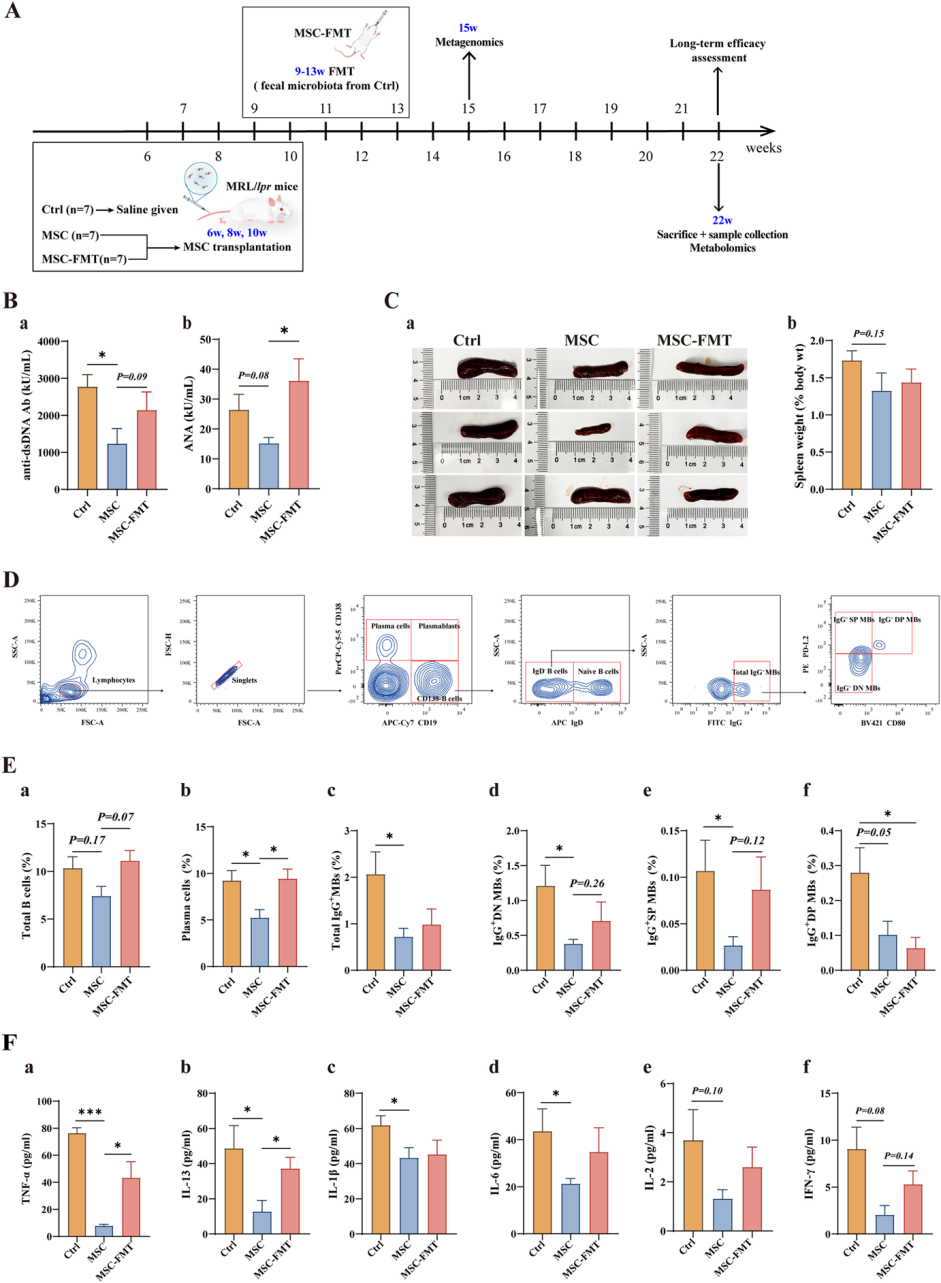
hUC-MSC transplantation reduces autoimmunity and inflammatory responses in MRL/*lpr* mice by week 22. **A** Experimental timeline schematic. **B** hUC-MSC transplantation reduces anti-double-stranded DNA (dsDNA) antibody levels (**a**) and antinuclear antibody (ANA) levels (**b**). **C** hUC-MSC transplantation ameliorates splenomegaly; representative spleen images from Ctrl, MSC, and MSC-FMT groups (**a**) and spleen weight/body weight percentage (**b**). **D** Peripheral blood flow cytometry gating strategy. **E** hUC-MSC transplantation reduces peripheral blood B cells. Frequencies of total B cells (**a**), plasma cells (**b**), total immunoglobulin (Ig)G^+^ memory B cells (MBs) (**c**), IgG^+^ CD80^−^ PD-L2^−^ double-negative memory B cells (DN MBs) (**d**), IgG^+^ CD80^−^ PD-L2^+^ single-positive memory B cells (SP MBs) (**e**), and IgG^+^ CD80^+^ PD-L2^+^ double-positive memory B cells (DP MBs) (**f**). **F** hUC-MSC transplantation reduces inflammatory cytokines. Plasma levels of tumor necrosis factor alpha (TNF-α) (**a**), interleukin (IL)-13 (**b**), IL-1β (**c**), IL-6 (**d**), IL-2 (**e**), and interferon gamma (IFN-γ) (**f**) in Ctrl, MSC, and MSC-FMT groups of lupus mice at week 22. *N* = 7 per group. *P < 0.05, ***P < 0.001

In addition to blood collection, the kidneys, spleen, lymph nodes, and colons were also harvested. The murine kidneys, spleens, and colons were carefully dissected into three distinct tissue pieces. One piece was snap-frozen in liquid nitrogen and stored at −80 °C for real-time polymerase chain reaction (RT-PCR) detection. Another piece was embedded in paraffin and sectioned into 3 μm thick slices for histopathological examinations. The third piece was embedded in optimal cutting temperature compound (OCT), frozen in liquid nitrogen, and sectioned into 5 μm thick slices for immunofluorescence (IF) analysis. A schematic diagram illustrating the experimental timeline is shown in Fig. 1A.

### Pathology assessment

For the pathological staining analysis, kidney and colon paraffin-embedded tissue sections (3 μm thick) were subjected to hematoxylin and eosin (H&E) staining. Histological analysis, conducted by a pathologist in a blinded manner, was based on clinical and pathological scores following the approach of our previous study [17]. Kidney sections were additionally stained with Masson’s trichrome, enabling quantitative assessment of fibrosis severity through analysis of collagen deposition. In total, ten randomly selected microscopic fields per sample were analyzed using ImageJ (ImageJ, Maryland, USA) to calculate the collagen-positive area percentage, with blue chromogen specifically indicating collagen fibers. Pathologic images of the kidneys and colons were captured using a VS200 light microscope and a BX43 light microscope (Olympus, Japan).

### Immunofluorescence (IF)

For the IF analysis, the frozen tissue blocks of kidneys, spleens, and colons were sectioned into 5 μm thick cryosections.

For the detection of glomerular immune complex deposition, renal frozen sections were blocked with 5% bovine serum albumin (BSA). Sections were incubated with Alexa Fluor 647-conjugated donkey anti-mouse IgG at a dilution of 1:400 and Alexa Fluor 488-conjugated rat anti-mouse complement 3 (C3) at a dilution of 1:100. Semiquantitative analysis of glomerular C3 deposition was performed using a scale of 0 to 3 (0 indicating negative, 1 indicating barely visible at high magnification, 2 indicating moderately visible, and 3 indicating clearly visible), as per a previous study [21].

For other target proteins, frozen sections were incubated with primary antibodies after blocking to analyze protein localization and expression. After washing, the sections were incubated with Alexa Fluor 594 or Alexa Fluor 488 secondary antibodies. Nuclei were stained with 4′,6-diamidino-2-phenylindole (DAPI).

Immunofluorescence images of the kidneys and spleens were captured using an Olympus VS200 fluorescence microscope (Olympus, Japan). Immunofluorescence images of the colons were captured using a Zeiss LSM 900 confocal laser scanning microscope (Carl Zeiss, Germany).

### Urine protein measurement

From week 6 to week 20, mice were individually placed in metabolic cages every 2 weeks. They were fasted overnight and given access to water for the collection of urine samples. After centrifugation (2000*g*, 4 °C, 10 min), the supernatant of urine samples was immediately stored at −80 °C. The concentration of proteinuria was detected using an autoanalyzer (Cobas8000, Roche, Switzerland), and the total 24-h urinary protein was calculated.

### Flow cytometry

Blood samples were collected from mice and centrifuged to separate cellular components from plasma. The cellular pellet was immediately resuspended in red blood cell (RBC) lysis buffer to lyse and remove red blood cells. After washing, the resulting peripheral blood mononuclear cells (PBMCs) were used for flow cytometry analysis. The PBMCs were incubated with purified anti-mouse CD16/32 antibody to prevent nonspecific antibody interactions. These samples were immunostained with APC-Cy7-conjugated anti-CD19, Percp-Cy5.5-conjugated anti-CD138, PE-conjugated anti-PD-L2/CD273, FITC-conjugated anti-IgG, APC-conjugated anti-IgD, and BV421-conjugated anti-CD80 antibodies. The peripheral blood flow cytometry gating strategy in MRL/*lpr* mice was performed as previously described (Fig. 1D) [22, 23]. Among the B cell subsets, CD19^−^ CD138^+^ plasma cells (PCs), CD19^+^ CD138^+^ plasmablasts (PBs), and CD138^−^ B cells could be gated. Additionally, CD19^+^ CD138^−^ IgD^−^ IgG^+^ B cells could be identified as total IgG^+^ memory B cells (MBs). These total IgG^+^ MBs could then be further classified as CD80^−^ PD-L2^−^ double-negative memory B cells (DN MBs), CD80^+^ PD-L2^+^ double-positive memory B cells (DP MBs), and CD80^−^ PD-L2^+^ single-positive memory B cells (SP MBs). The catalog numbers of the antibodies and reagents used, along with their respective concentrations, are meticulously detailed in Supplementary Table S2. Data were analyzed using FlowJo 10.8.1 software (FlowJo LLC, Ashland, Wilmington, DE, USA). The FlowAI plugin was used to remove abnormal events and generate normal events [24].

### Enzyme-linked immunoassay (ELISA) assay

Plasma levels of antinuclear antibodies (ANA) and anti-double-stranded DNA (dsDNA) antibodies were measured using a Mouse Anti-Nuclear Antibodies Total Ig ELISA Kit (cat. no. 5210, Alpha Diagnostic, USA) and a Mouse Anti-dsDNA Antibodies Total Ig ELISA Kit (cat. no. 5110, Alpha Diagnostic, USA), respectively, following the manufacturer’s instructions. For each assay, approximately 10 μL of plasma was used. The ANA assay utilized a dilution ratio of 1:100, while the anti-dsDNA assay utilized a dilution ratio of 1:4000.

### Determination of plasma cytokine levels

Plasma sample processing was outsourced to Guangzhou Juyan Biological Co., Ltd. (Guangzhou, China) for plasma cytokine level detection. For each sample, 50 μL of plasma was used for the multiplex cytokine assay, as recommended by the manufacturer. Plasma levels of inflammatory cytokines, including tumor necrosis factor alpha (TNF-α), interferon gamma (IFN-γ), interleukin (IL)-1β, IL-2, IL-6, and IL-13, were assayed using a Milliplex^®^ MAP kit (cat. no. MHSTCMAG-70 K, Millipore, Billerica, MA, USA), following the manufacturer’s instructions.

### Determination of plasma biochemical parameters

Plasma creatinine levels and plasma urea nitrogen levels were measured using a plasma creatinine assay kit (cat. no. C011-2-1, Jiancheng Bio, China) and a blood urea nitrogen (BUN) assay kit (cat. no. C013-2-1, Jiancheng Bio, China), respectively. For each assay, 10 μL of plasma was used without dilution.

### Assessment of spleen enlargement

The 22-week-old MRL/*lpr* mice were fasted for 12 h, and their total body weight was measured before sacrifice. Spleens were harvested, and their gross appearance and weights were recorded. The spleen index was calculated as the spleen weight divided by the body weight.

### Metagenomic sequencing

The 15-week fecal samples were processed and sequenced using metagenomic techniques at Wuhan MetWare Biotechnology Co., Ltd. (Wuhan, China). For each group, four mice were randomly selected using a completely randomized numerical table method. The experiment had three main steps: sample testing, library construction, and sequencing. During sample testing, two methods were used to assess the DNA samples. First, DNA degradation and potential contamination were checked using 1% agarose gels. Second, DNA concentration was measured with the Qubit^®^ dsDNA Assay Kit in the Qubit^®^ 2.0 Fluorometer. Samples with OD values between 1.8 and 2.0 and DNA content above 1 μg were chosen for library construction. During library construction, 1 μg of DNA from each sample was used as input material. The NEBNext^®^ Ultra™ DNA Library Prep Kit for Illumina was used to create sequencing libraries according to the manufacturer’s instructions. The following primers were used for the construction of the sequencing libraries:

1. 5′ universal adapter primer:
  - Full-length primer: 5′-AATGATACGGCGACCACCGAGATCTACACTCTTTCCCTACACGACGCTCTTCCGATCT-3′
  - p5 primer binding to the flowcell: 5′-AATGATACGGCGACCACCGAGATCTACAC-3′
  - Read1 primer: 5′-TCTTTCCCTACACGACGCTCTTCCGATCT-3′
2. 3′ adapter primer (index1):
  - Full-length primer with index: 5′-CAAGCAGAAGACGGCATACGAGATCGTGATGTGACTGGAGTTCAGACGTGTGCTCTTCCGATCT-3′
  - p7 primer binding to the flowcell: 5′-CAAGCAGAAGACGGCATACGAGAT-3′
  - Read2 primer: 5′-GTGACTGGAGTTCAGACGTGTGCTCTTCCGATCT-3′

Index codes were added to assign sequences to each sample. DNA samples were sonicated to 350 bp, followed by end-polishing, A-tailing, and ligation with full-length adaptors for Illumina sequencing. Standard PCR amplification was performed, and PCR products were purified using the AMPure XP system. The size distribution of the libraries was analyzed with the Agilent 2100 Bioanalyzer, and quantification was conducted using real-time PCR. For sequencing, the index-coded samples were clustered with a cBot Cluster Generation System following the manufacturer’s instructions. After cluster generation, the libraries were sequenced on an Illumina NovaSeq platform, generating paired-end reads. Metagenomic data analysis begins with preprocessing sequencing results and metagenome assembly, followed by gene prediction, abundance analysis, species annotation, and annotation with a common functions database. According to previous reports, species annotation was conducted using the DIAMOND software [25]. Principal component analysis (PCA) is a dimensionality reduction technique that uses variance decomposition to simplify multidimensional data, extracting the most significant elements and structures. Intergroup comparisons at the genus and species levels were performed using GraphPad Prism 9.0 software with one-way analysis of variance (ANOVA). Linear discriminant analysis effect size (LEfSe) analysis identifies species biomarkers with significant differences between groups; it first detects differential species between groups using the rank sum test, and then it performs dimensionality reduction and evaluates the impact of the differential species with LDA, yielding the LDA score. In this study, an LDA score above 2 was used to distinguish distinct gut microbiota among the groups. Using tools from the Cloudtutu website (https://www.cloudtutu.com/), we performed a random forest analysis on bacterial species. The top seven important gut microbiota at the species level were ranked on the basis of a percent increase in mean squared error (%IncMSE) calculated using random forest. Tools from OmicStudio (https://www.omicstudio.cn/tool) were used for analyzing taxonomy abundance Circos diagrams, and Wekemo Bioincloud (https://www.bioincloud.tech) was used for the Spearman correlation network diagram.

### Widely targeted metabolomics

The 22-week plasma samples were processed and analyzed for widely targeted metabolomics at Wuhan MetWare Biotechnology Co., Ltd. For each group, four mice were randomly selected using a completely randomized numerical table method. For each sample, 100 μL of plasma was used without dilution. The samples were thawed from a −80 °C freezer and ground in liquid nitrogen (20 mg each). After adding 400 µL of 70% methanol/water internal standard, the mixture was vortexed and centrifuged at 16,260*g* and 4 °C for 10 min. The supernatant was collected for analysis after being stored at −20 °C for 30 min and centrifuged again at 16,260*g* and 4 °C for 3 min. Metabolite detection and identification were conducted using liquid chromatography–mass spectrometry (LC–MS) with an electrospray ionization (ESI) source at Wuhan MetWare Biotechnology Co., Ltd. Data analysis was performed using the Analyst 1.63 software and the MetWare database. Using the R package (V3.5.1) and MetaboAnalystR (V1.0.1), orthogonal partial least squares discrimination analysis (OPLS-DA) was performed to obtain variable importance in prediction (VIP) values. For two-group analysis, differential metabolites were identified using VIP (VIP > 1) and *P*-value (*P*-value < 0.05, Student’s *t*-test). The data were log-transformed (log2) and mean-centered before OPLS-DA. To avoid overfitting, a permutation test with 200 permutations was conducted. Identified metabolites were annotated using the KEGG Compound database and mapped to the KEGG Pathway database. Significantly enriched pathways were identified using a hypergeometric test’s *P*-value for a given list of metabolites. Spearman’s rank correlation analysis and redundancy analysis (RDA) were performed using OmicStudio tools and Wekemo Bioincloud, respectively.

### Culture of HT-29 cells

Human colon epithelial cancer cells (HT-29) were purchased from the National Collection of Authenticated Cell Cultures (cat. no. TCHu103, Shanghai, China). HT-29 cells were cultured in RPMI-1640 medium (GE Healthcare Bio Science, Hyclone) with 10% fetal bovine serum (FBS) (cat. no. abs974-500ml, Absin, China) and 1% penicillin/streptomycin (cat. no. 15140122, Gibco, USA) at 5% CO_2_ and 37 °C. To investigate the effects of specific metabolites on *AHR* gene expression and downstream pathway activation, HT-29 cells were treated with three key metabolites identified through metabolic profiling analysis. These metabolites—3-indoleacrylic acid (cat. no. GC1970; GLPBIO, USA), 1-acetylindole (cat. no. GD01258; GLPBIO, USA), and tributyrin (cat. no. GC61349; GLPBIO, USA)—were selected on the basis of their significant upregulation in mice treated with MSCs and their potential role in modulating AHR activity. Each metabolite was tested at concentrations of 0, 10, 50, 100, and 200 μM for 24 h. All experiments were performed in triplicate to ensure reproducibility. After treatment, cells were harvested for RNA extraction and subsequent gene expression analysis via RT-PCR, as detailed below.

### Real-time PCR (RT-PCR)

RNA was extracted from colonic tissues of MRL/*lpr* mice and HT-29 cell lysates using the RNAiso Plus reagent (cat. no. 9109; TaKaRa, Japan) following the protocol provided by the manufacturer. Subsequently, the RNA was converted into cDNA with the aid of the ChamQ Blue Universal SYBR RT-PCR Master Mix (cat. no. Q312-02; Vazyme Biotech Co. Ltd, China). For gene expression analysis, quantitative real-time PCR was conducted on LightCycler^®^ 480 Instrument II (Roche), employing HiScript III RT SuperMix for RT-PCR (+gDNA wiper) (cat. no. R323-01; Vazyme Biotech Co. Ltd, China) as the fluorescence dye. The expression levels of the genes of interest were standardized against *ACTB*, employed as a reference gene, and the relative quantification was determined using the 2^−(ΔΔCT)^ method. Primer sequences are detailed in Supplementary Table S3.

### Molecular docking

To perform molecular docking of metabolites (as ligands) with the AHR protein (as the receptor), the three-dimensional structures of the ligands were downloaded from PubChem (https://pubchem.ncbi.nlm.nih.gov/). The crystal structure of the AHR protein in PDB format was then downloaded from the RCSB Protein Data Bank (https://www.rcsb.org/). Using PyMOL software (Version 2.4.0), the ligands and water molecules were removed from the AHR protein, and the cleaned structure was saved in PDB format. Molecular docking of the selected ligands with the AHR protein was performed using the CB-Dock2 server (https://cadd.labshare.cn/cb-dock2/php) [26].

### Statistical analyses

Data are expressed as mean ± standard error of the mean (SEM) and were analyzed using the GraphPad Prism 8.0.2 statistical software (GraphPad Software, Inc., San Diego, CA, USA). The Shapiro–Wilk test was used to determine whether the data followed a normal or nonnormal distribution. For normally distributed data, Bartlett’s test evaluated variance homogeneity; one-way ANOVA was applied when variances were equal, while the Brown–Forsythe and Welch ANOVA tests were used for unequal variances. Nonnormally distributed data were analyzed using the Kruskal–Wallis nonparametric test. Statistical significance was defined as *P* < 0.05.

## Results

### hUC-MSC transplantation reduces the severity of autoimmunity and inflammatory response in MRL/*lpr* mice

At week 22, the MSC group showed a significant reduction in anti-dsDNA autoantibody levels compared with the Ctrl group (*P* < 0.05; Fig. 1B, panel a) and a trend toward decreased ANA antibodies (*P* = 0.08; Fig. 1B, panel b). However, the MSC-FMT group showed a significant increase in ANA compared with the MSC group (*P* < 0.05; Fig. 1B, panel b) and an upward trend in anti-dsDNA autoantibodies (*P* = 0.09; Fig. 1B, panel a). We observed a trend for decreased spleen volume and spleen weight/bodyweight percentage in the MSC group compared with the Ctrl and MSC-FMT groups (*P* = 0.15; Fig. 1C, panel a, b). These results suggest that hUC-MSC transplantation significantly reduces autoantibody production and ameliorates splenomegaly in lupus mice. Transplanting fecal microbiota from lupus mice with active disease weakens the therapeutic effects of hUC-MSCs on autoantibody levels and splenomegaly. This suggests that the inhibitory effect of hUC-MSCs on SLE autoimmunity may be closely related to gut microbiota balance.

We speculated whether MSC transplantation affects the differentiation of B cell subsets, including plasma cells (PCs) and memory B cells (MBs) (Fig. 1D, E). PCs and MBs participate in SLE occurrence and progression. At week 22, the MSC group showed a significant decrease in the frequency of PCs, total IgG^+^MBs, IgG^+^DN MBs, and IgG^+^SP MBs (*P* < 0.05; Fig. 1E, panels b–e). Additionally, compared with the Ctrl group, the MSC group showed a downward trend in the frequency of IgG^+^DP MBs (*P* = 0.05; Fig. 1E, panel f) and total B cells (*P* = 0.17; Fig. 1E, panel a). Conversely, the MSC-FMT group showed a significant increase in the frequency of PCs compared with the MSC group (*P* < 0.05; Fig. 1E, panel b). The MSC-FMT group also showed an upward trend in the frequency of total B cells (*P* = 0.07; Fig. 1E, panel a) and IgG^+^SP MBs (*P* = 0.12; Fig. 1E, panel e). These results indicate that hUC-MSC transplantation may attenuate peripheral blood B cell proliferation, which could be associated with reduced plasma autoantibody levels. Transplanting fecal microbiota from lupus mice during the active disease phase may diminish the inhibitory effect of hUC-MSCs on B cells, consistent with findings on autoantibodies.

Plasma levels of TNF-α, IL-13, IL-1β, and IL-6 were significantly decreased in the MSC group at week 22 compared with the Ctrl group (*P* < 0.001, *P* < 0.05, *P* < 0.05, *P* < 0.05, respectively; Fig. 1F, panels a–d). There was also a decreasing trend in the levels of IL-2 and IFN-γ (*P* = 0.10, *P* = 0.08, respectively; Fig. 1F, panels e, f).

The MSC-FMT group showed significantly increased levels of TNF-α (*P* < 0.05; Fig. 1F, panel a) and IL-13 (*P* < 0.05; Fig. 1F, panel b) compared with the MSC group, with a trend of increased IFN-γ (*P* = 0.14; Fig. 1F, panel f).

We assessed the distribution of plasma cells (PCs) in the spleen using immunofluorescence. The MSC group showed a significant decrease in fluorescence expression of CD19^−^ CD138^+^ PCs compared with the Ctrl group following hUC-MSCs transplantation (*P* < 0.05, Supplementary Fig. S1, A–B). However, the MSC-FMT group showed an increasing trend in fluorescence expression of PCs compared with the MSC group. These findings suggest that transplanting fecal microbiota from lupus mice with active disease may attenuate the suppressive effect of hUC-MSC treatment on antibody-forming B cells.

These results indicate that early hUC-MSC transplantation in MRL/*lpr* mice with SLE can effectively lower subsequent plasma cytokine levels and reduce the proportion of splenic plasma cells. Transplanting fecal microbiota from lupus mice with active disease diminishes the therapeutic effects of hUC-MSCs on autoimmunity.

### hUC-MSC transplantation ameliorates lupus nephritis in MRL/*lpr* mice

**Fig. 2:**
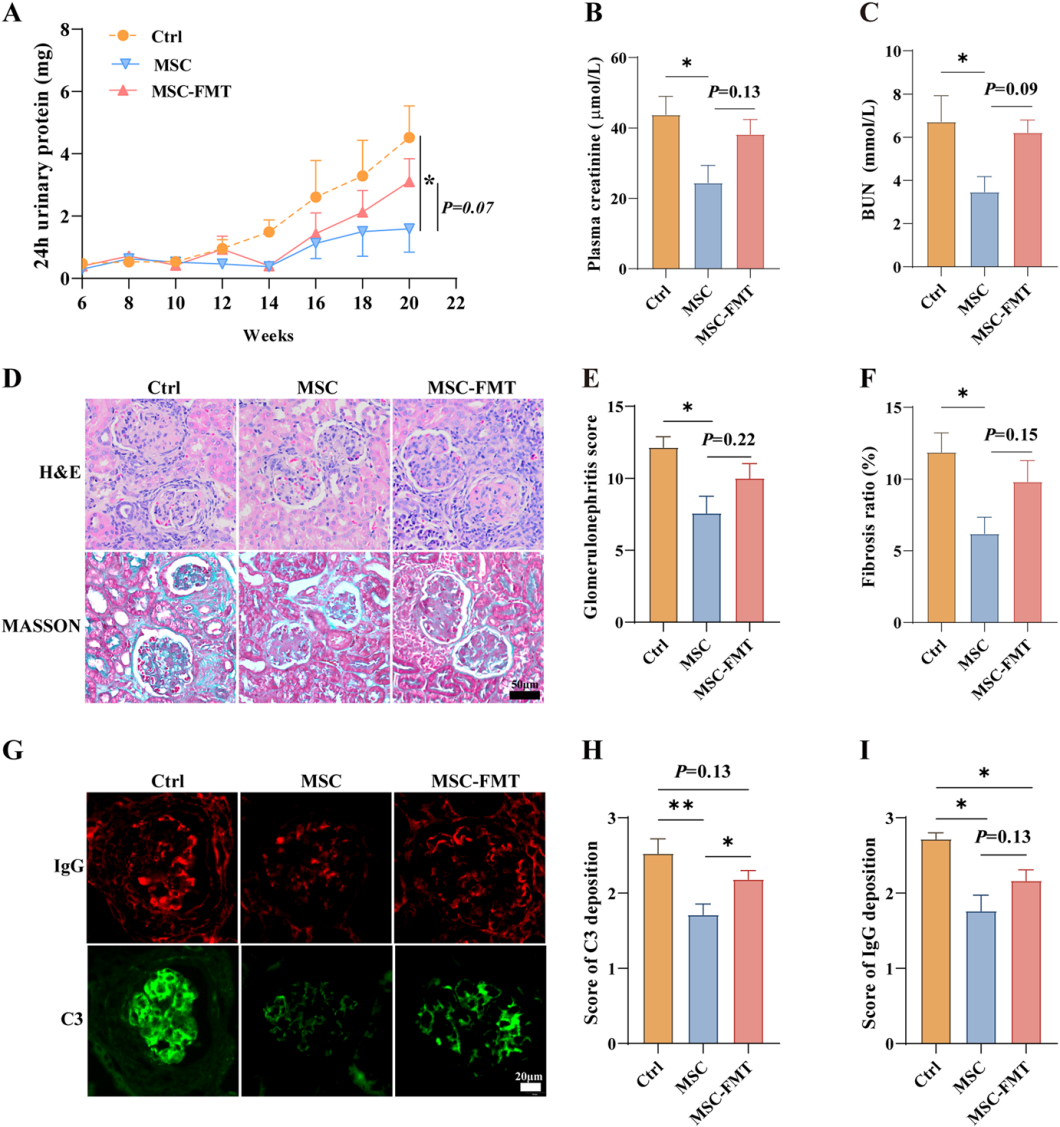
hUC-MSC transplantation improves lupus nephritis in MRL/*lpr* mice at week 22. **A** Line plot of 24-h urinary protein levels in MRL/*lpr* mice from weeks 6 to 20. **B** Plasma creatinine levels in MRL/*lpr* mice at week 22. **C** BUN levels in MRL/*lpr* mice at week 22. **D** Representative images of renal pathological staining (H&E and Masson) across all groups at week 22 (scale bar = 50 μm). **E** Glomerulonephritis scores of renal lesions. **F** Quantitative analysis of renal fibrosis (% blue staining). **G** Representative images of renal immunofluorescence deposition of IgG and complement C3 in all groups at week 22 (scale bar = 20 μm). **H** Quantitative analysis of glomerular C3 deposition. **I** Quantitative analysis of glomerular IgG deposition. *N* = 7 per group. *P < 0.05, **P < 0.01

**Fig. 3:**
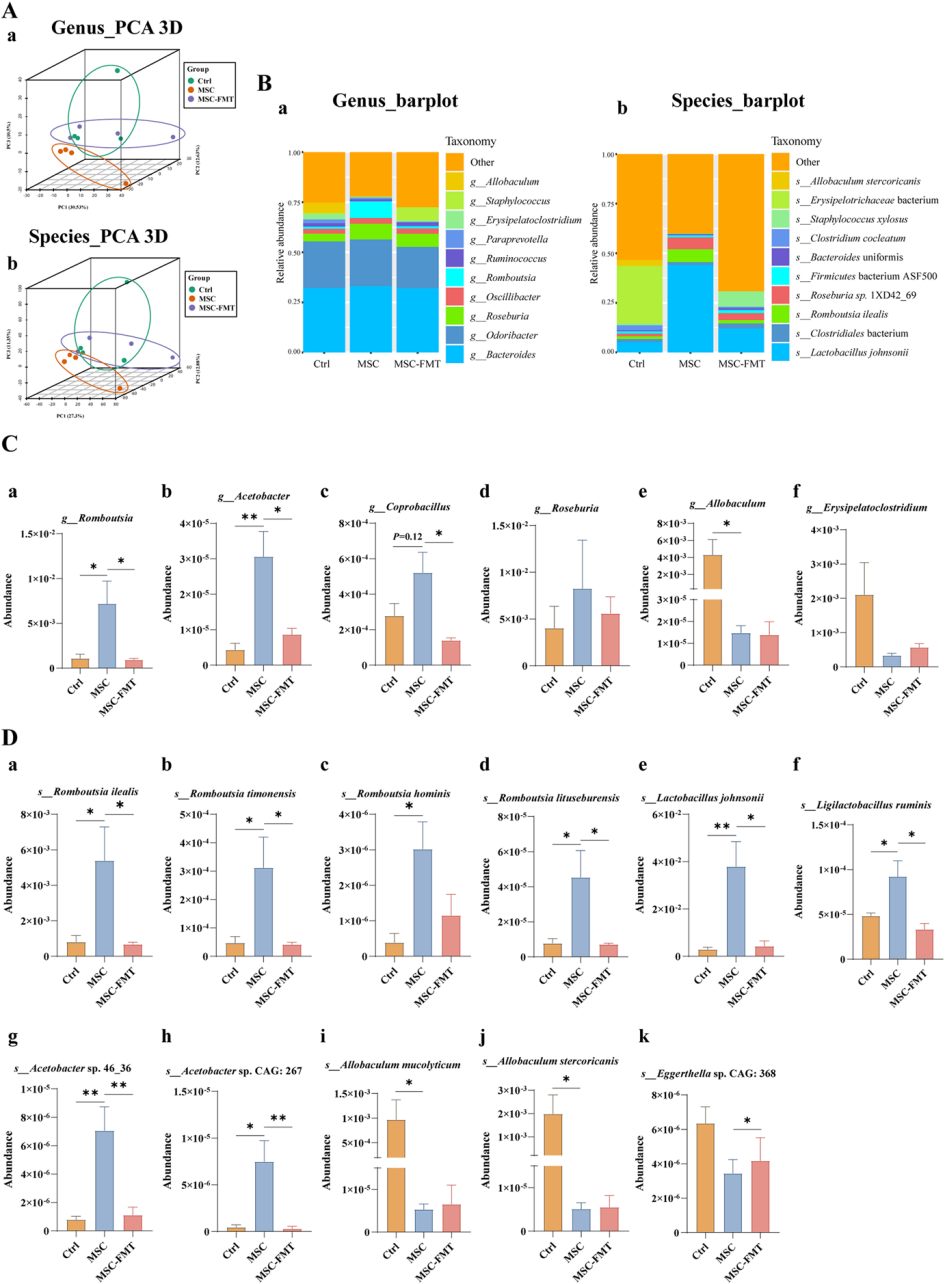
Metagenomic analysis of fecal samples from MRL/*lpr* mice at week 15. **A**, **B** Three-dimensional principal component analysis (3D-PCA) (**A**) and taxonomic abundance barplots (**B**) at genus (**a**) and species (**b**) levels among all groups. **C** Statistical analysis of microbiota at the genus level among all groups. **D** Statistical analysis of microbiota at the species level among all groups. *N* = 4 per group. *P < 0.05, **P < 0.01

We next assessed the levels of urinary protein (Fig. 2A). The 24-h urinary protein graph (weeks 6–20) indicates that the MSC group had significantly reduced urinary protein compared with the Ctrl group (*P* < 0.05, Fig. 2A). The MSC-FMT group showed an increasing trend in urinary protein compared with the MSC group (*P* = 0.07, Fig. 2A). Next, we detected plasma creatinine and BUN as indicators of renal function. Results showed that hUC-MSC transplantation significantly improved plasma creatinine (*P* < 0.05, Fig. 2B) and BUN levels (*P* < 0.05, Fig. 2C) compared with the Ctrl group. In the MSC-FMT group, plasma creatinine and BUN levels tended to be higher than those in the MSC group (Fig. 2B, C). We further examined renal damage in MRL/*lpr* mice. The MSC group showed improvement in renal pathological injury and fibrosis severity compared with the Ctrl group (*P* < 0.05, Fig. 2D–F). However, the MSC-FMT group showed no significant improvement in renal pathological injury and glomerular fibrosis (Fig. 2D–F). At week 22, IgG and C3 deposition were significantly reduced in the kidneys of the MSC group compared with the Ctrl group (*P* < 0.01, *P* < 0.05, respectively; Fig. 3G–I). The MSC-FMT group had reduced C3 and IgG deposition compared with the Ctrl group, but it was higher than in the MSC group (*P* < 0.05, *P* = 0.13, respectively; Fig. 3G–I).

In summary, these results suggest that early hUC-MSC transplantation during SLE can significantly reduce immune complex formation and ameliorate lupus nephritis. FMT (as seen in the MSC-FMT group) partially reduced the protective effects of hUC-MSCs.

### hUC-MSC transplantation improves gut microbiota dysbiosis in MRL/*lpr* mice

The 3D-PCA results show distinct sample separation among the Ctrl, MSC, and MSC-FMT groups at the genus and species levels (Fig. 3A, panels a, b). The taxonomic abundance barplots indicate significant differences in gut microbiota among the groups at the genus and species levels (Fig. 3B, panels a, b). The abundances of *Romboutsia* and *Acetobacter* were significantly higher in the MSC group than the Ctrl and MSC-FMT groups at the genus level (*P* < 0.05, *P* < 0.01, respectively; Fig. 3C, panels a, b). In the MSC group, the genus *Coprobacillus* showed an increasing trend (*P* = 0.12, Fig. 3C, panel c). The MSC group showed a significant reduction in the abundance of genus *Allobaculum* compared with the Ctrl group (*P* < 0.05, Fig. 3C, panel e).

**Fig. 4:**
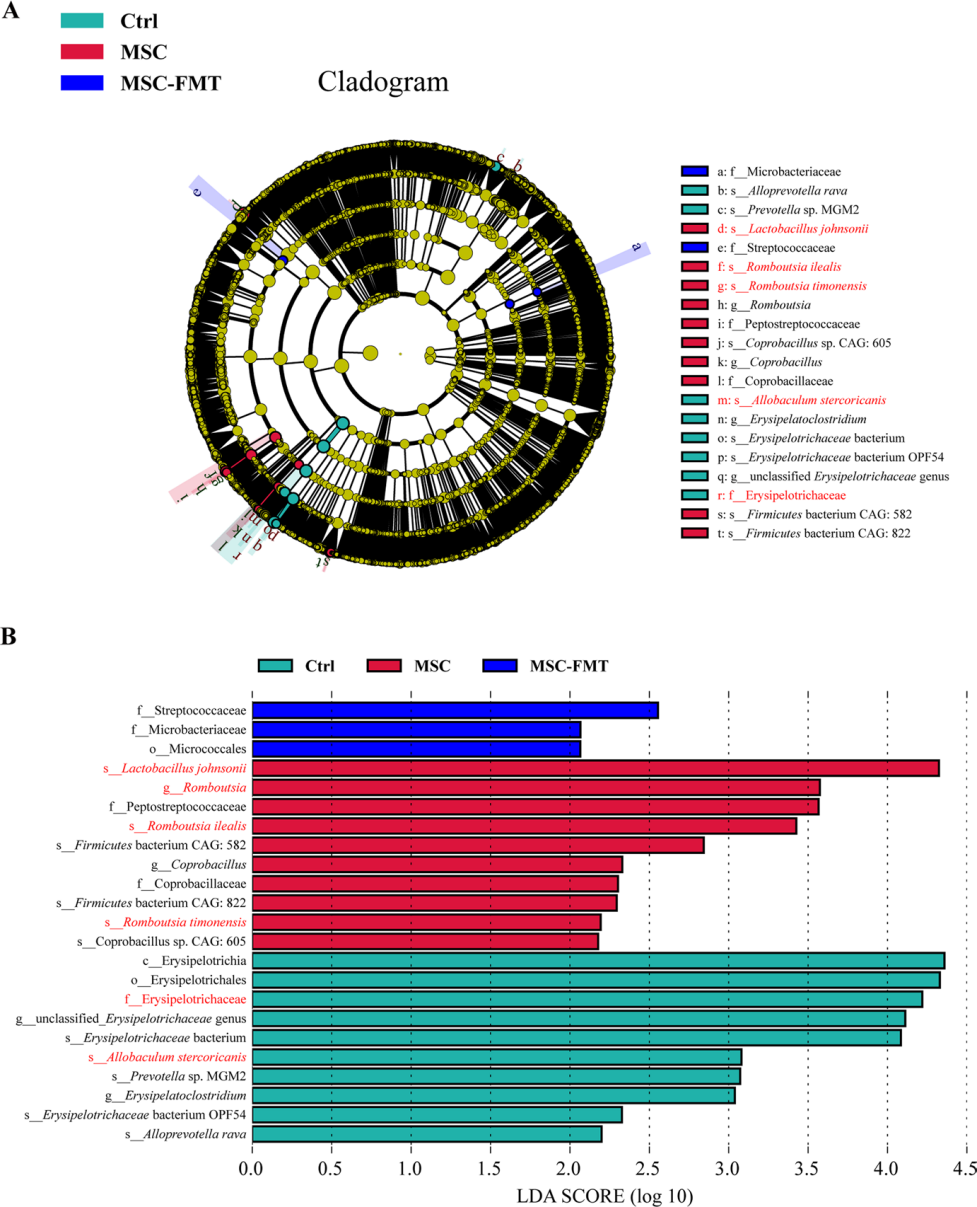
LEfSe analysis of fecal metagenomics from MRL/*lpr* mice at week 15. **A**, **B** Cladograms and bar plots were generated using linear discriminant analysis (LDA) effect size (LEfSe) to determine significantly different microbiota taxa among all groups (LDA > 2, *P* < 0.05). Node diameter in the cladograms is proportional to the relative abundance of the taxonomic units. Green indicates taxa enriched in Ctrl, red in MSC, and blue in MSC-FMT. *N* = 4 per group

The abundance of commensal bacteria, including* Romboutsia ilealis (R. ilealis), Romboutsia timonensis (R. timonensis), Romboutsia hominis (R. hominis), and Romboutsia lituseburensis (R. lituseburensis)* (*P* < 0.05, Fig. 3D, panels a–d), as well as *Lactobacillus johnsonii (L. johnsonii), Ligilactobacillus ruminis*, *Acetobacter* sp. 46_36, and *Acetobacter* sp. CAG: 267 (*P* < 0.01, *P* < 0.05, *P* < 0.01, *P* < 0.05, respectively; Fig. 3D, panels e–h), significantly increased in the MSC group compared with the Ctrl and MSC-FMT groups. Conversely, the abundance of *Allobaculum mucolyticum* (*A. mucolyticum*) and *Allobaculum stercoricanis* (*A. stercoricanis*) in the MSC group significantly decreased (*P* < 0.05, Fig. 3D, panels i, j). The abundance of *Eggerthella* sp. CAG: 368 significantly increased in the MSC-FMT group compared with the MSC group (*P* < 0.05, Fig. 3D, panel k). Similar results were obtained from the LEfSe analysis. The abundance of *A. stercoricanis* (family: Erysipelotrichaceae) was significantly enriched in the Ctrl group, whereas *R. ilealis*, *R. timonensis*, and *L. johnsonii* were significantly enriched in the MSC group (Fig. 4A, B). Beneficial bacteria such as *R. ilealis* and *L. johnsonii* significantly increased, while pathogenic bacteria such as *A. mucolyticum* and *A. stercoricanis* significantly decreased. The gut microbiota of the MSC-FMT group was similar to that of the Ctrl group.

These results indicate that fecal microbiota transplantation from lupus mice with active disease might reverse the early regulatory effects of hUC-MSC transplantation on intestinal microbiota homeostasis in SLE.

### hUC-MSC transplantation modulates key gut microbiota and interspecies interactions in MRL/*lpr* mice

**Fig. 5:**
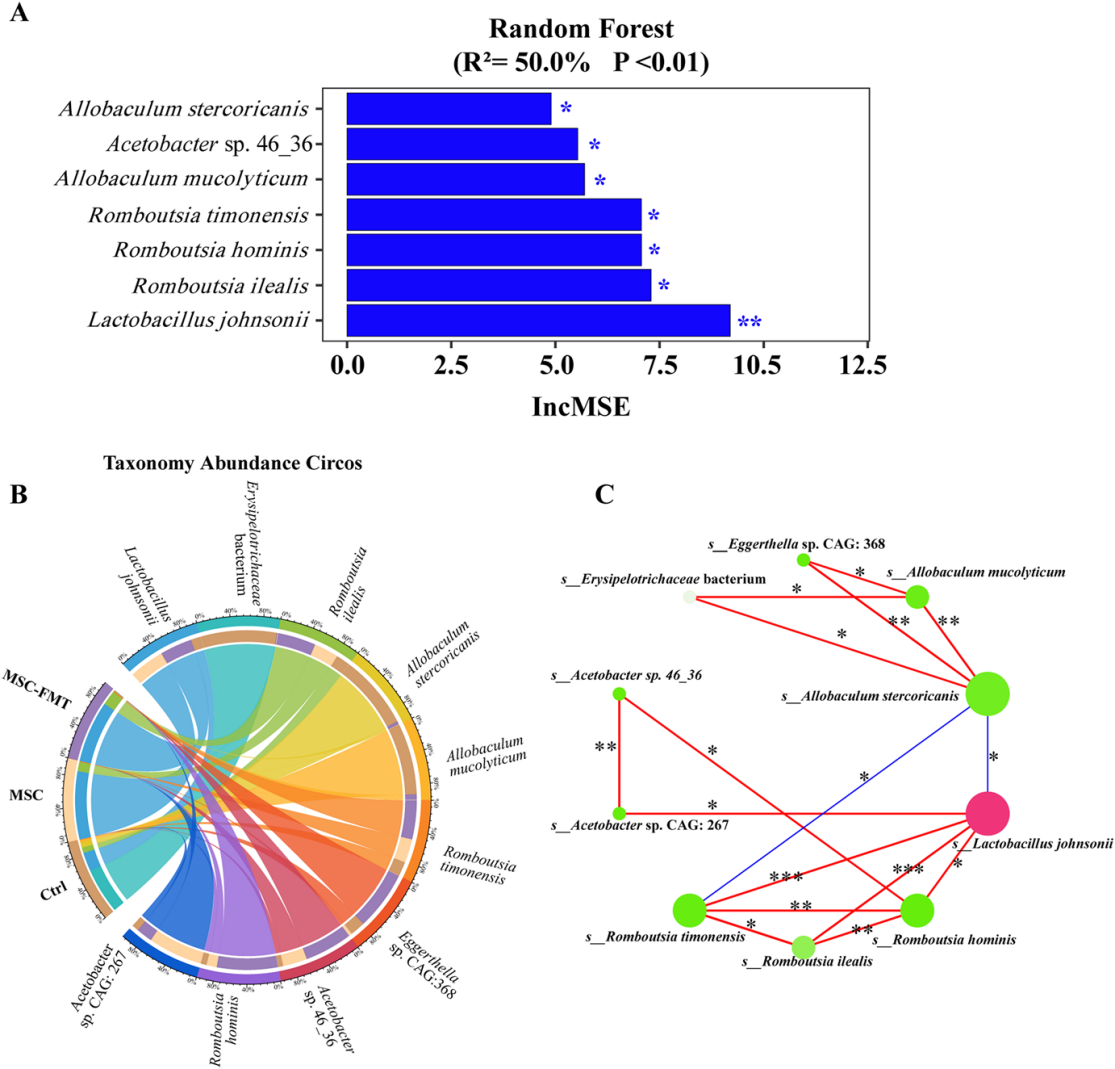
hUC-MSC transplantation modulates key gut microbiota and their interactions in 15-week-old MRL/*lpr* mice. **A** Random forest modeling was used to compare species profiles. **B** Taxonomic abundance Circos plot of species among all groups. The Circos taxonomy abundance diagram consists of two concentric circles showing sample groups and species classifications. The left side depicts sample groups color-coded in the outer ring. The inner ring displays the percentage of each species within the samples. On the right, the outer ring color bar represents different species, while the inner ring bar length indicates the species proportion, consistent with sample group colors. **C** Network diagram showing Spearman correlation analysis among key gut microbiota at the species level. Circular nodes represent species abundance, with larger nodes indicating higher abundance. Red lines indicate positive correlations, blue lines indicate negative correlations, and thicker lines denote stronger correlations. *P < 0.05, **P < 0.01, ***P < 0.001

We used a random forest model to compare the species spectra of Ctrl, MSC, and MSC-FMT groups to identify key bacteria regulated by hUC-MSC (Fig. 5A). The key bacteria were ranked by the percent increase in mean squared error (%IncMSE). The most significant bacteria were *L. johnsonii*, *R. ilealis*, *R. hominis*, *R. timonensis*, *A. mucolyticum*, *Acetobacter* sp. 46_36, and *A. stercoricanis*, in that order (Fig. 5A). The Circos taxonomy abundance diagram displayed the relationships between the abundance of key species and samples from different groups (Fig. 5B). Samples for *L. johnsonii*, *R. ilealis*, *R. hominis*, *R. timonensis*, *Acetobacter* sp. 46_36, and *A. stercoricanis* were predominantly from the MSC and MSC-FMT groups (Fig. 5B). *A. mucolyticum*, *A. stercoricanis*, and *Eggerthella* sp. CAG: 368 were primarily found in Ctrl group samples. The network diagram of the Spearman correlation analysis illustrated the interrelationships among key species (Fig. 5C). *L. johnsonii* positively correlated with *R. ilealis* (*P* < 0.01), *R. timonensis* (*P* < 0.01), *R. hominis* (*P* < 0.05), and *Acetobacter* sp. CAG: 267 (*P* < 0.05), suggesting that these commensal bacteria may act synergistically (Fig. 5C). Additionally, *L. johnsonii* and *R. timonensis* negatively correlated with *A. stercoricanis* (*P* < 0.05, Fig. 5C), indicating a potential antagonistic relationship between these bacteria and *A. stercoricanis*. *A. stercoricanis* is significantly positively correlated with *A. mucolyticum* (*P* < 0.01), *Eggerthella* sp. CAG: 368 (*P* < 0.01), and *Erysipelotrichaceae* bacterium (*P* < 0.05), suggesting that these bacteria may act in concert (Fig. 5C). This complex network reflects the intricate relationships within the gut microbiota.

In summary, hUC-MSC transplantation ameliorates gut microbiota dysbiosis by upregulating beneficial bacteria and downregulating pathogenic bacteria. The complex interaction network among the gut microbiota may significantly influence the therapeutic effects of hUC-MSC transplantation. Transplantation of fecal microbiota from mice with active lupus disease may diminish the therapeutic effects of hUC-MSC treatment in MRL/*lpr* mice.

### hUC-MSC transplantation improved plasma metabolic abnormalities in MRL/*lpr* mice

**Fig. 6:**
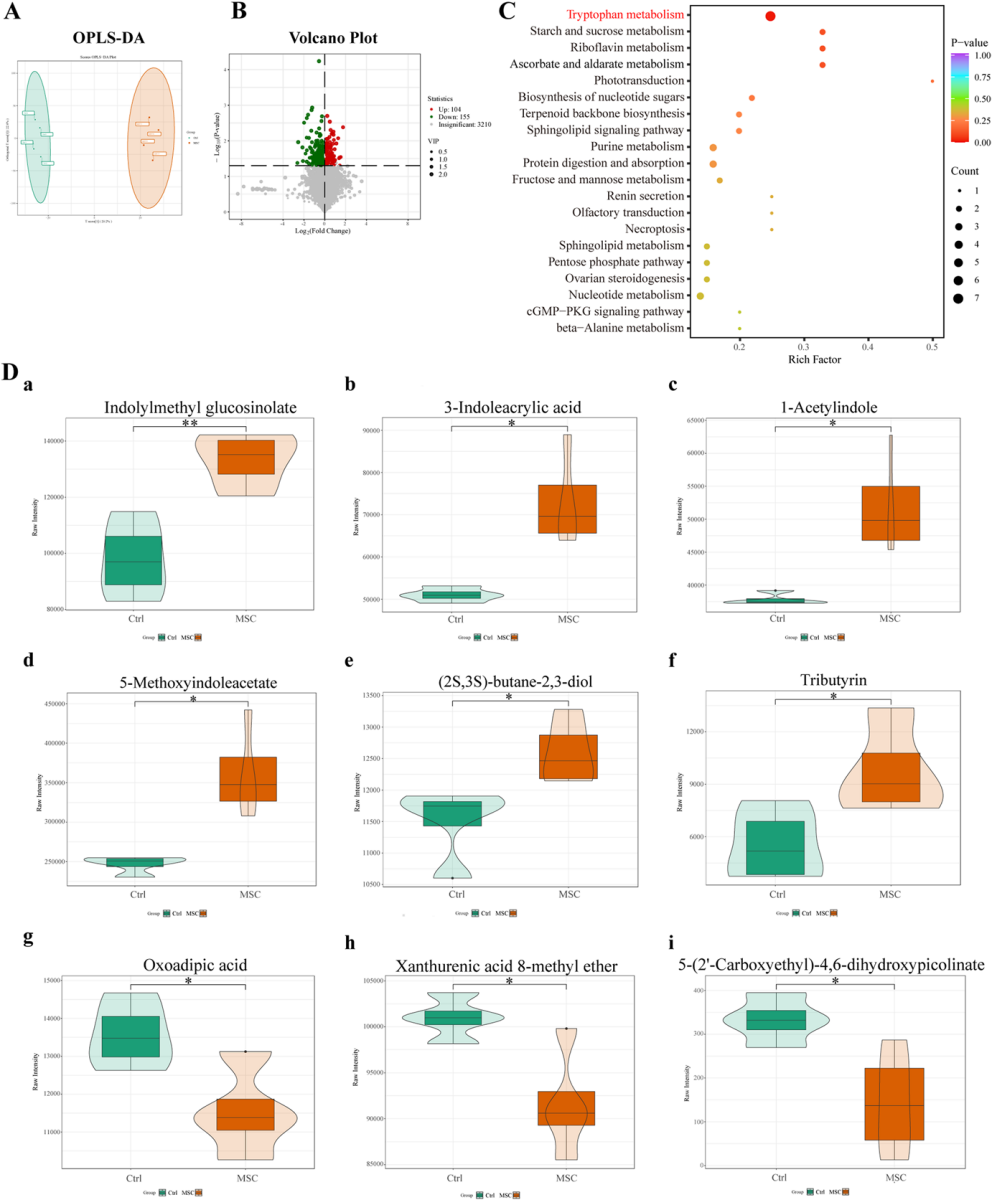
Metabolomic analysis of plasma samples from MRL/*lpr* mice at week 22. **A** OPLS-DA score chart for the Ctrl (green) and MSC (red) mouse groups. **B** Volcano plot of differential metabolites. Differential metabolites were selected on the basis of VIP > 1 and *P*-value < 0.05. **C** KEGG enrichment analysis bubble plot. Bubble size represents the number of differential metabolites in each pathway, while bubble color indicates the *P*-value for each pathway. The Trp metabolism pathway shows the highest enrichment. **D** Violin plots of specific metabolites in correlation analysis. The box in the center of the violin plot represents the interquartile range, and the thin black lines represent the 95% confidence interval. The central black line indicates the median, and the outer shapes depict data distribution density. *N* = 4 per group

**Fig. 7:**
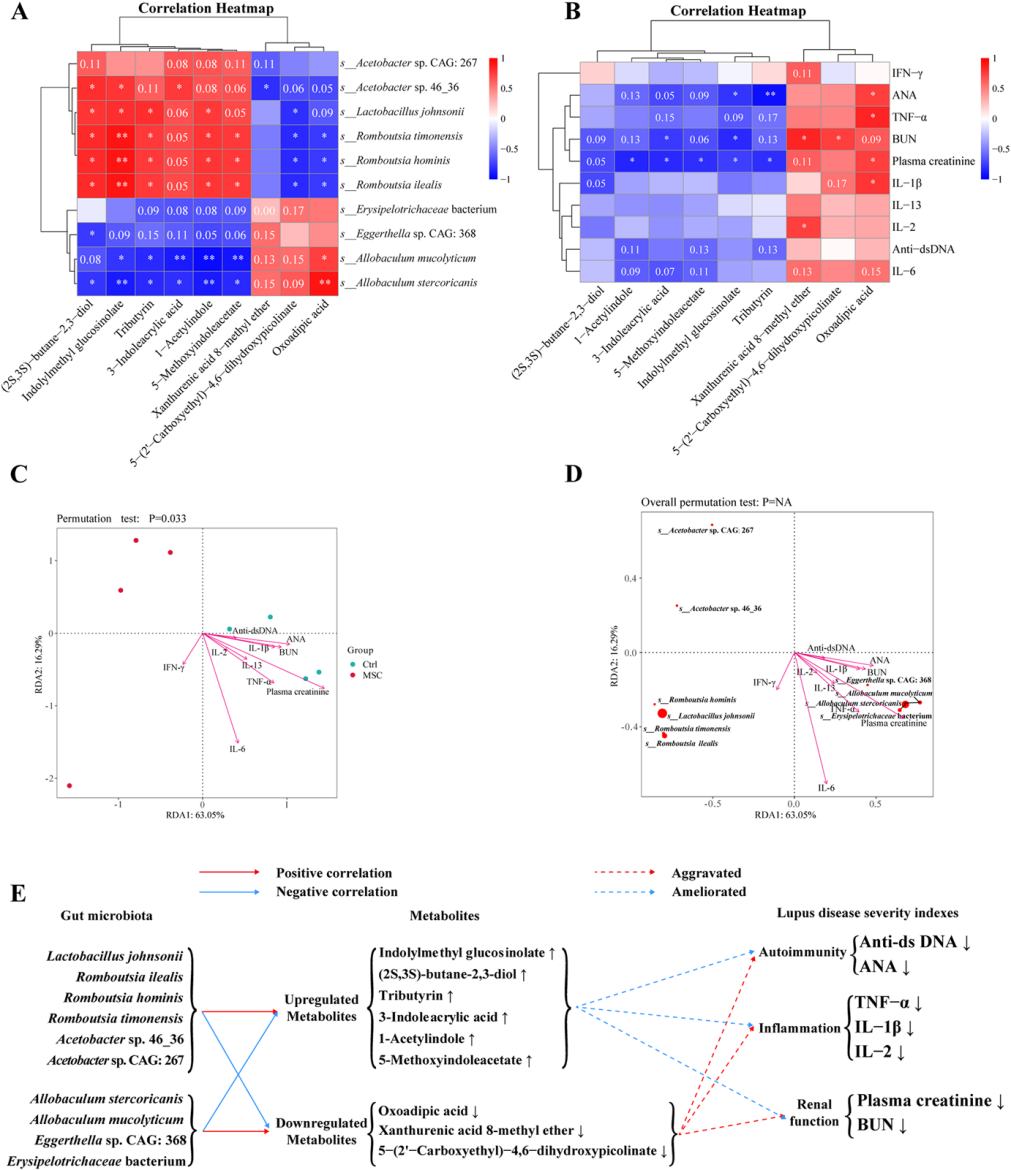
Correlation analysis of gut microbiota with differential metabolites and lupus disease severity indices. **A**, **B** Spearman’s rank correlation coefficient heatmap. Correlation analysis between microbiota species abundance and specific metabolites (**A**). Correlation analysis between specific metabolites and lupus disease severity indices (**B**). R-values are indicated by colors: red squares for positive correlation, blue for negative, and darker shades for stronger correlations. The numbers in the heatmap cells indicate *P*-values. *N* = 4 per group. *P < 0.05, **P < 0.01. **C, D** RDA analysis between microbiota species abundance and disease severity indices. Distribution of samples and lupus disease severity indices (**C**). The distribution of microbiota species and disease severity indices in RDA analysis are shown (**D**). The angle between the arrow and the ordination axis indicates correlation: acute for positive and obtuse for negative. *N* = 4 per group. **E** Schematic representation of species abundance association with specific metabolites and disease severity indices

OPLS-DA analysis showed a clear separation between the Ctrl and MSC groups (Fig. 6A). The volcano plot displayed 104 upregulated and 155 downregulated metabolites in the MSC group (Fig. 6B). Spearman correlation analysis was conducted to investigate the potential functional association between gut microbiota and plasma metabolites (Fig. 7A). To investigate the involvement of metabolites in lupus pathogenesis, Spearman correlation analysis was conducted between these metabolites and lupus disease severity indexes (Fig. 7B). Redundancy analysis (RDA) plots and schematic showed significant associations between specific gut microbiota, metabolites, and lupus disease severity indices (Fig. 7C–E).

The results showed high enrichment for “tryptophan (Trp) metabolism” (Fig. 6C, Supplementary Fig. S2, A–B), indicating significant plasma Trp metabolism after hUC-MSC transplantation. Disturbances in Trp metabolism may contribute to the mechanism of lupus autoimmune activation. Indole derivatives associated with Trp metabolism, such as indolylmethyl glucosinolate, 3-indoleacrylic acid, 1-acetylindole, and 5-methoxyindoleacetate, significantly increased in the MSC group (*P* < 0.01, *P* < 0.05, *P* < 0.05, *P* < 0.05, respectively; Fig. 6D, panels a–d). Elevated levels of these indole derivatives showed varying degrees of negative correlation with plasma autoantibodies, proinflammatory cytokines, and renal function indices (Fig. 7B). Specifically, indolylmethyl glucosinolate significantly negatively correlated with plasma ANA, creatinine, and BUN levels (*P* < 0.05, Fig. 7B). 1-acetylindole, 3-indoleacrylic acid, and 5-methoxyindoleacetate significantly negatively correlated with plasma creatinine levels (*P* < 0.05, Fig. 7B). The MSC group showed significant reductions in downstream metabolites of kynurenine, including xanthurenic acid 8-methyl ether, 5-(2′-carboxyethyl)-4,6-dihydroxypicolinate, and oxoadipic acid (*P* < 0.05, Fig. 6D, panels g–i). Oxoadipic acid showed significant positive correlations with plasma ANA, IL-1β, TNF-α, and creatinine, while Xanthurenic acid 8-methyl ether was positively correlated with IL-2 and BUN (*P* < 0.05, Fig. 7B).

Short-chain fatty acids (SCFAs) produced by gut microbiota, such as butyrate, improve gut barrier function and have therapeutic effects on autoimmune diseases [27]. Herein, (2S,3S)-butane-2,3-diol (2,3-BD), a butyrate derivative, significantly increased in the MSC group (*P* < 0.05, Fig. 6D, panel e); 2,3-BD was annotated in the KEGG pathway for “Butanoate metabolism” (Supplementary Fig. S2A). We observed a significant increase in tributyrin, a butyric acid precursor, in the MSC group (*P* < 0.05, Fig. 6D, panel f). Tributyrin, consisting of a glycerol backbone and three butyrate molecules, shows higher stability and produces more butyrate in the gut than other forms of butyrate [28]. Our results indicate that tributyrin significantly negatively correlates with antinuclear antibodies (ANA) and plasma creatinine levels (*P* < 0.01, *P* < 0.05, respectively, Fig. 7B). Elevation of tributyrin may reduce autoantibodies in SLE, potentially ameliorating lupus symptoms.

Enriched species in the MSC group, including *L. johnsonii*, *R. ilealis*, *R. timonensis*, *R. hominis*, and *Acetobacter* sp. 46_36, showed positive correlations with beneficial metabolites such as indolylmethyl glucosinolate and tributyrin, and negative correlations with oxoadipic acid and 5-(2′-carboxyethyl)-4,6-dihydroxypicolinate (Fig. 7A). RDA results showed that *L. johnsonii*, *R. ilealis*, *R. timonensis*, *R. hominis*, and *Acetobacter* sp. 46_36 were negatively correlated with plasma autoantibodies (ANA and anti-dsDNA), inflammatory cytokines (TNF-α and IL-1β), and renal function indices (plasma creatinine and BUN) (Fig. 7D, E). The Trp metabolism pathway was associated with *L. johnsonii* and *R. ilealis* (Supplementary Table S1). These results suggest that *L. johnsonii* could improve Trp metabolic disturbances in lupus mice by increasing indole derivatives and decreasing kynurenine derivatives. While the overall impact was positive, the MSC-FMT group’s results indicated that FMT somewhat reduced the protective benefits of hUC-MSC.

### hUC-MSC transplantation may enhance intestinal microbiota-related metabolites and ameliorate intestinal injury in MRL/*lpr* mice through AHR activation in intestinal epithelial cells

*Lactobacillus* can produce Trp metabolites, including 3-indoleacrylic acid, which ameliorates intestinal inflammation and injury by binding to and activating AHR, thereby alleviating rheumatoid arthritis [29].

**Fig. 8:**
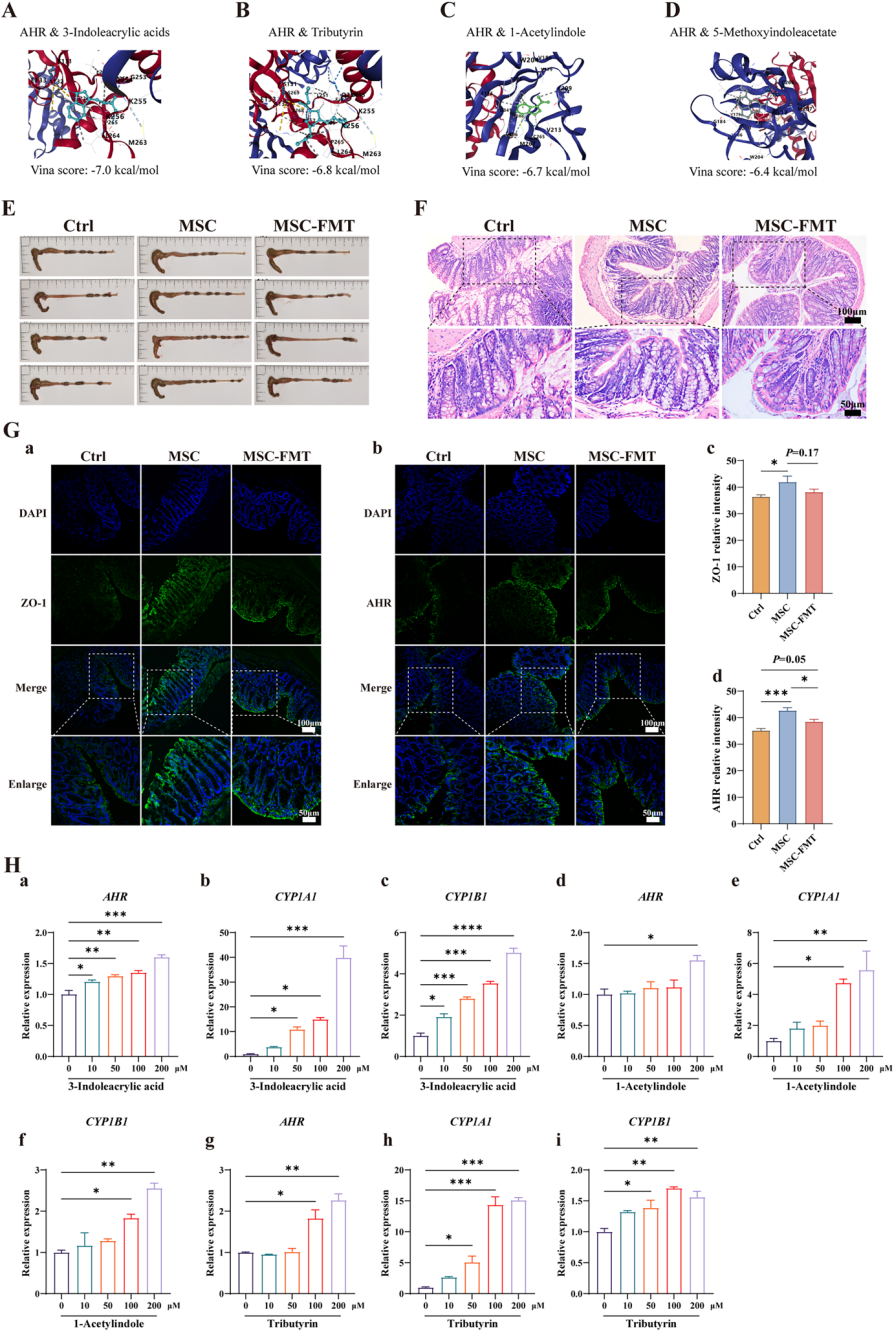
hUC-MSC transplantation enhances gut microbiota-related metabolites that may ameliorate intestinal injury in MRL/*lpr* mice through the activation of AHR in intestinal epithelial cells. **A**–**D** Molecular docking diagrams of four metabolites with the aryl hydrocarbon receptor (AHR): 3-indoleacrylic acid (**A**), tributyrin (**B**), 1-acetylindole (**C**), and 5-methoxyindoleacetate (**D**). **E** Representative images of colons from the Ctrl, MSC, and MSC-FMT groups. **F** Representative images of colonic H&E staining in each group. Scale bars are 100 µm (overview) and 50 µm (magnified insets). **G** Intestinal zonula occludens 1 (ZO-1) and AHR fluorescence images and quantitative analysis results. Representative immunofluorescence images of ZO-1 (**a**) and AHR (**b**) in colons from Ctrl, MSC, and MSC-FMT-transplanted mice. Scale bars are 100 µm (overview) and 50 µm (magnified insets). Quantitative analysis of ZO-1 levels (**c**). Quantitative analysis of AHR levels (**d**). *N* = 7 per group. *P < 0.05, ***P < 0.001. **H** Key metabolite treatments enhance *AHR* gene expression and downstream pathway activation in HT-29 cells. RT-PCR quantification of relative gene expression for *AHR*, *CYP1A1*, and *CYP1B1* in HT-29 cells after 24-h exposure to 3-indoleacrylic acid (**a–c**), 1-acetylindole (**d–f**), and tributyrin (**g–i**) at 0, 10, 50, 100, and 200 μM concentrations. *N* = 4 per group. *P < 0.05, **P < 0.01, ***P < 0.001, ****P < 0.0001

To determine whether Trp-related metabolites that were upregulated following hUC-MSC transplantation can interact with AHR, molecular docking was performed using the CB-DOCK2 tool. The CB-DOCK2 framework utilizes the Vina score to evaluate the efficacy of molecular docking and predict the binding mode and affinity of ligands at protein binding sites. A lower Vina score signifies a more stable ligand–protein interaction, indicating a superior docking result. The molecular docking Vina scores for 3-indoleacrylic acid, tributyrin, 1-acetylindole, and 5-methoxyindoleacetate were −7.0, −6.8, −6.7, and −6.4 kcal/mol, respectively (Fig. 8A–D). This suggests that the binding stability of these metabolites with AHR, from strongest to weakest, is as follows: 3-indoleacrylic acid being the most stable and 5-methoxyindoleacetate the least. The *Lactobacillus* metabolite 5-methoxyindoleacetate activates AHR and contributes to ameliorating intestinal inflammation and maintaining intestinal immune homeostasis [30]. Consequently, it is hypothesized that 3-indoleacrylic acid, tributyrin, 1-acetylindole, and 5-methoxyindoleacetate may bind to and activate AHR, potentially improving intestinal damage and barrier function.

Compared with the control group, the colon length of mice in the MSC group increased at week 22 (Fig. 8E), with reduced inflammatory cell infiltration and increased goblet cells (Fig. 8F). Mice in the MSC-FMT group exhibited shortened colon length and increased intestinal inflammatory cell infiltration at 22 weeks (Fig. 8E, F).

The expression of tight junction protein zonula occludens 1 (ZO-1) protein in the colon was subsequently assessed using immunofluorescence. Compared with the control group, ZO-1 expression in the colon increased in the MSC group (*P* < 0.05, Fig. 8G, panels a, c). A decreasing trend in ZO-1 was observed in the MSC-FMT group compared with the MSC group (*P* = 0.17, Fig. 8G, panels a, c). This suggests that hUC-MSC transplantation can increase ZO-1 expression in the intestine, facilitating the repair of intestinal damage. The expression of AHR protein in the colon was also assessed using immunofluorescence. Compared with the control group, AHR expression in colonic epithelial cells increased in the MSC group following hUC-MSC transplantation (*P* < 0.001, Fig. 8G, panels b, d). A significant decrease in AHR was observed in the MSC-FMT group compared with the MSC group (*P* < 0.05, Fig. 8G, panels b, d).

We used RT-PCR to further examine the relative expression levels of the *Ahr*, *Cyp1a1*, and *Cyp1b1* genes in the colon tissues of mice from each group (as shown in Supplementary Fig. S3). The data indicated that, compared with the Ctrl group, the expression of the colonic *Ahr* gene and its downstream *Cyp1a1* and *Cyp1b1* genes was significantly upregulated in the MSC-transplanted group (*P* < 0.001, Supplementary Figure S3, A-C). When comparing the MSC-FMT group with the MSC group, the expression levels of *Ahr*, *Cyp1a1*, and *Cyp1b1* were significantly reduced (*P* < 0.05, Supplementary Fig. S3, A–C). Therefore, MSC transplantation can promote the expression of *Ahr*, *Cyp1a1*, and *Cyp1b1* genes in the colon of MRL/*lpr* mice, while FMT can diminish this promotional effect.

In our experiments, we identified 3-indoleacrylic acid, 1-acetylindole, and tributyrin as metabolites with significant molecular docking affinity to the AHR. To evaluate their impact on intestinal epithelial cells, we treated HT-29 cells with these metabolites at concentrations ranging from 0 to 200 μM for 24 h. Using RT-PCR, we quantified the relative gene expression of *AHR* and its downstream targets, *CYP1A1* and *CYP1B1*.

Our results indicated that all three metabolites induced a dose-dependent increase in *AHR* gene expression in HT-29 cells (Fig. 8H, panels a, d, g). This suggests that these metabolites are capable of binding to and activating AHR, which is a key regulator in intestinal homeostasis. Consistent with AHR activation, the gene expression of *CYP1A1* and *CYP1B1* was also significantly elevated, indicating the activation of the AHR signaling pathway (Fig. 8H).

In conclusion, hUC-MSC transplantation effectively elevates metabolites associated with beneficial bacteria, such as 3-indoleacrylic acid, 1-acetylindole, and tributyrin, which, upon binding and activation of the AHR, ameliorate intestinal damage in MRL/*lpr* mice. Additionally, the reduced expression of AHR and ZO-1 in the MSC-FMT group underscores the role of the gut microbiome in lupus-prone mice, suggesting that alterations in the gut microbiota can decrease the expression of these proteins, leading to intestinal injury.

## Discussion

Lupus mice exhibit early onset gut dysbiosis [8], and MSC-based therapies are typically used for treating severe or refractory SLE [31]. In our recent study, we demonstrated that early MSC intervention inhibits B cell subset differentiation and ameliorates lupus symptoms in mice within 4 weeks [17]. This study investigates whether early MSC therapy can extend therapeutic efficacy to 12 weeks by modulating gut microbiota and metabolism. Our study demonstrated that hUC-MSC transplantation early in SLE has long-term therapeutic effects in lupus-prone MRL/*lpr* mice, improving gut microbiota dysbiosis and plasma abnormalities in Trp and butyrate metabolism. However, FMT (MSC-FMT group) somewhat diminished these protective effects.

In this study, hUC-MSC transplantation early in SLE significantly decreased plasma levels of proinflammatory cytokines. Reduced B cell subsets in peripheral blood decreased autoantibodies, as autoantibody production is associated with AFCs, including PBs and PCs [32]. MBs differentiate into AFCs to produce antibodies and enter germinal centers for B cell immunity [33]. Consistent with previous reports [33, 34], our FCM results showed a reduction in memory B cell subsets (DN MBs, DP MBs, and SP MBs) and PCs in the MSC group. hUC-MSC transplantation significantly improved urinary protein, plasma creatinine, and BUN levels in lupus mice, reduced immune complex and complement deposition, and ameliorated pathological damage and fibrosis. In contrast, the MSC-FMT group had elevated autoantibodies and inflammatory cytokines, increased PCs, exacerbated renal damage, and accumulated immune complexes.

Lupus mice exhibit early onset gut microbiota dysbiosis compared with normal mice [35]. Therefore, we speculated that early MSC transplantation in SLE would alleviate lupus progression by regulating gut microbiota dysbiosis in MRL/*lpr* mice. Abnormalities in Trp metabolism may contribute to lupus autoimmune activation [36], with gut microbiota playing a significant role in producing Trp metabolites [37]. To investigate the mechanism underlying hUC-MSC transplantation in SLE treatment, we conducted fecal metagenomic sequencing at week 15 and plasma metabolomic sequencing at week 22 in MRL/*lpr* mice.

In metagenomic sequencing, *Romboutsia* and *Acetobacter* were significantly increased in the MSC group at the genus level. Moreover, compared with the Ctrl and MSC-FMT groups, the abundance of *R. ilealis*, *R. timonensis*, *R. hominis*, *Acetobacter* sp. 46_36, and *Acetobacter* sp. CAG:267 was significantly higher in the MSC group at the species level. *L. johnsonii* alleviates impaired gut barrier function, reduces IgG2a levels, and improves lupus nephritis in a hormone-dependent manner [38]. Consistent with previous findings [39], we found a significant increase in *L. johnsonii* in the MSC group. *Romboutsia* and *Acetobacter* produce SCFAs [40, 41], which are crucial for colonocyte energy and gut barrier integrity [42]. Dietary sodium butyrate can improve gut microbiota dysbiosis and lupus nephritis in lupus mice [43]. In this study, species enriched in the MSC group, such as *L. johnsonii*, *R. ilealis*, and *R. timonensis*, were positively correlated with butanoate metabolites (Tributyrin and 2,3-BD). Tributyrin supplementation improves intestinal dysbiosis, increases SCFAs, and improves intestinal barrier function and inflammation [44]. Our results suggest that tributyrin is negatively correlated with ANA and plasma creatinine.

Metabolomic sequencing indicated that “tryptophan metabolism” was a significantly enriched pathway. *Lactobacillus* produces 3-indoleacrylic acid, which activates AHR, alleviates intestinal inflammation, and mitigates rheumatoid arthritis [29]. The binding stability of 3-indoleacrylic acid, tributyrin, 1-acetylindole, and 5-methoxyindoleacetate to AHR decreased in that order. 5-methoxyindoleacetate activates AHR, improves intestinal inflammation, and maintains immune homeostasis by regulating the Th17/Treg cell balance [30]. AHR activation is crucial for intestinal health and prevents inflammation and pathogen translocation [45–48]. Trp metabolites activate AHR to improve intestinal inflammation via multiple pathways. AHR activation enhances IL-22 expression for mucosal homeostasis [49]. AHR activation also affects gut microbiota composition, promoting the growth of beneficial bacteria such as *Lactobacillus*, which produce AHR agonists, creating a positive feedback mechanism that enhances intestinal health [48].

In this study, hUC-MSC transplantation increased beneficial bacteria (such as *L. johnsonii*, *R. ilealis*, *R. timonensis*, and *R. hominis*) and plasma Trp (3-indoleacrylic acid) and butyrate (tributyrin) metabolites. RT-PCR analysis showed that MSC transplantation significantly increased colonic *Ahr*, *Cyp1a1*, and *Cyp1b1* gene expression compared with controls, while FMT reduced this upregulation. Furthermore, indole derivatives (3-indoleacrylic acid, 1-acetylindole) and tributyrin enhanced *AHR* gene expression and activation in HT-29 cells, upregulating downstream target genes *CYP1A1* and *CYP1B1*.

These metabolites may improve intestinal damage and barrier function by activating the AHR. Intestinal inflammation is characterized by shortened colon length, increased inflammatory cell infiltration, and decreased goblet cells. hUC-MSC transplantation in early SLE improved colon length, reduced inflammatory cell infiltration, and increased goblet cells, while FMT inhibited these beneficial effects. AHR activation can enhance ZO-1 expression and localization by upregulating the Notch1 signaling pathway, restoring the structural integrity of intestinal tight junctions [50, 51]. hUC-MSC transplantation increased AHR and ZO-1 expression in colon epithelial cells of lupus mice, while FMT inhibited this regulation.

This study primarily investigated the role of MSCs in increasing beneficial bacteria and improving SLE, without exploring the specific mechanisms of MSC modulation of the gut microbiota. MSCs are multipotent stem cells with antimicrobial characteristics, acting through direct bactericidal activity and modulation of the host’s innate and adaptive immune cells [52]. The specific mechanisms by which MSCs facilitate the colonization of beneficial bacteria may relate to creating a favorable gut microenvironment. For example, MSCs produce antimicrobial peptides that selectively target pathogens [53], promote the survival and proliferation of intestinal epithelial cells, upregulate tight junction molecules, and maintain intestinal integrity [54]. Additionally, immunoregulatory probiotics such as *Lactobacillus rhamnosus* can amplify the immunomodulatory effects of MSCs in a lupus mouse model, indicating a synergistic therapeutic potential between *Lactobacillus* and MSCs [55].

We propose that MSC transplantation is effective in treating early SLE and may play a role in disease prevention. MSC treatment may reduce memory B cell subsets, thereby decreasing autoantibodies and improving SLE disease progression. However, MSCs may also indirectly improve immune cell function to delay SLE progression. Our study suggests that gut microbiota may be a mechanism through which MSCs indirectly modulate immune cell function, potentially playing a pivotal role in SLE treatment. However, our study has limitations. The small sample size and metabolic heterogeneity may have affected our findings, necessitating larger sample sizes in future studies. Additionally, MSC efficacy needs enhancement, and the animal model used cannot fully replicate the human response. Future analyses will involve humanized lupus model mice and clinical studies. Finally, the specific mechanisms of MSC modulation of gut microbiota and AHR activation promoting ZO-1 expression remain unclear.

## Conclusions

Our research underscores the partial mechanisms of hUC-MSC therapy in SLE, particularly the modulation of the gut microbiota–Trp–AHR axis. This intervention increases the abundance of beneficial bacteria such as *L. johnsonii* and *R. ilealis* and upregulates plasma Trp metabolites, which enhances their binding affinity to AHR and upregulates the expression of its downstream target genes *Cyp1a1* and *Cyp1b1*. The subsequent activation of AHR promotes ZO-1, crucial for intestinal repair. However, FMT (seen in the MSC-FMT group) reduced the protective benefits of hUC-MSC to some extent. This sequence of events reduces plasma autoantibodies and inflammatory cytokines, improves LN, and ultimately, slows lupus disease progression. Therefore, MSC transplantation may have promising long-term effects in SLE treatment by modulating gut microbiota and the gut microbiota–Trp–AHR axis and may serve as a novel therapeutic strategy for the early course of SLE.

## Supplementary Information

Additional file 1.

## Data Availability

The datasets used and/or analyzed during the current study are available from the corresponding author on reasonable request.

## Abbreviations

MSCs: Mesenchymal stem cells
hUC-MSCs: Human umbilical cord-derived mesenchymal stem cells
SLE: Systemic lupus erythematosus
Ctrl: Control
FMT: Fecal microbiota transplantation
AHR: Aryl hydrocarbon receptor
ZO-1: Zonula occludens 1
LN: Lupus nephritis
FBS: Fetal bovine serum
RBCs: Red blood cells
PBMCs: Peripheral blood mononuclear cells
PCs: Plasma cells
MBs: Memory B cells
ANA: Antinuclear antibodies
Anti-dsDNA: Anti-double-stranded DNA
DN MBs: CD80^−^ PD-L2^−^ double-negative memory B cells
DP MBs: CD80^+^ PD-L2^+^ double-positive memory B cells
SP MBs: CD80^−^ PD-L2^+^ single-positive memory B cells
TNF-α: Tumor necrosis factor alpha
IFN-γ: Interferon gamma
IL: Interleukin
PAS: Periodic acid–Schiff
H&E: Hematoxylin and eosin
BSA: Bovine serum albumin
SCFAs: Short-chain fatty acids
Trp: Tryptophan
PBS: Phosphate-buffered saline
IF: Immunofluorescence
