# Supplementary material for: Exploring the role of gut microbiota modulation in the long-term therapeutic benefits of early MSC transplantation in MRL/lpr mice
Source: Cell Mol Biol Lett. 2025 Apr 18;30:49. doi: 10.1186/s11658-025-00716-8 (PMC12007202; doi:10.1186/s11658-025-00716-8)
Supplement: Supplementary file 1 — Additional file 1. [file 11658_2025_716_MOESM1_ESM.docx]

SUPPLEMENTAL INFORMATION

**Quanren Pan ^1†^, Fengbiao Guo ^1†^, Jiaxuan Chen ^1†^, Haimin Huang ^1^, Yanyan Huang ^1^,**

**Shuzhen Liao** **^1^, Zengzhi Xiao ^1^, Xi Wang ^1^, Liuyong You ^2^, Lawei Yang ^1^, Xuemei Huang ^3^, Haiyan Xiao ^4^,** **Hua-Feng Liu ^1*^, Qingjun Pan ^1, 2, 5 *^**

^1^ Guangdong Provincial Key Laboratory of Autophagy and Major Chronic Non-communicable Diseases, Department of Nephrology, Affiliated Hospital of Guangdong Medical University, Zhanjiang, China

^2^ Department of Clinical Laboratory, State Key Laboratory of Respiratory Disease, The First Affiliated Hospital of Guangzhou Medical University, Guangzhou, China

^3^ Department of Anesthesiology, First people's hospital of Foshan, Foshan, Guangdong, China

^4^ Department of Cellular Biology and Anatomy, James and Jean Culver Vision Discovery Institute, Medical College of Georgia, Augusta University, Augusta, GA, United States

^5^ Clinical Research and Experimental Center, Affiliated Hospital of Guangdong Medical University, Zhanjiang, China

^†^ These authors contributed equally.

**^*^**Correspondence**:** liuhf@gdmu.edu.cn; stilwapan@gmail.com

**Supplementary information includes:**

**Supplementary Figures S1-S3.**

**Supplementary Tables S1-S3.**

**Supplementary Figures**


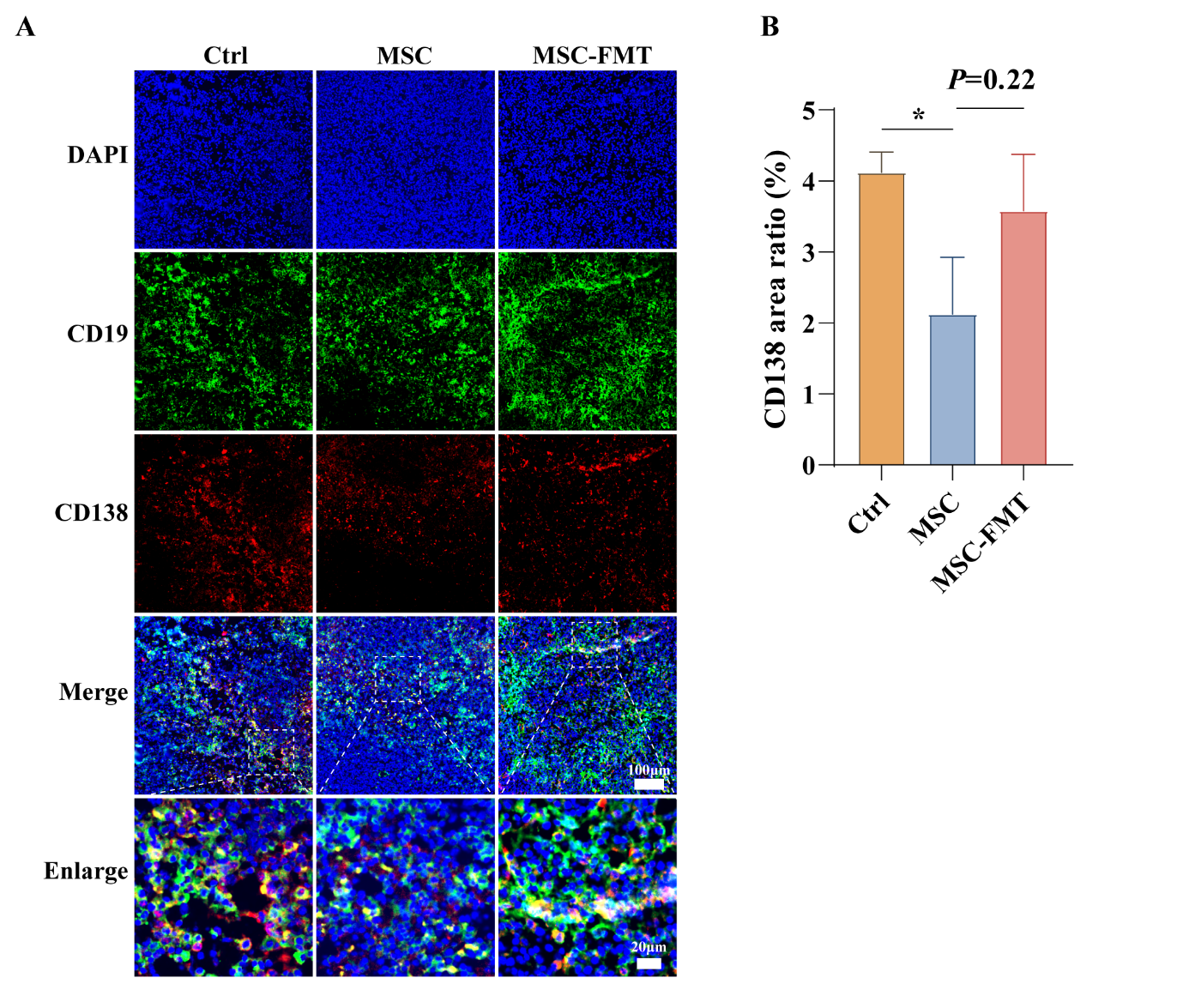


**Supplementary Figure S1.**  **hUC-MSC transplantation reduces splenic plasma cells in MRL/*lpr* mice at week 22.** (A) Representative immunofluorescence images of plasma cells (CD19^-^CD138^+^ PCs) in Ctrl, MSC, and MSC-FMT mice. Green fluorescence indicates CD19, and red indicates CD138. Scale bars: 100 µm (overview) and 20 µm (magnified insets). (B) Comparative analysis of the CD138-positive area ratio to total spleen area in lupus mice at week 22.


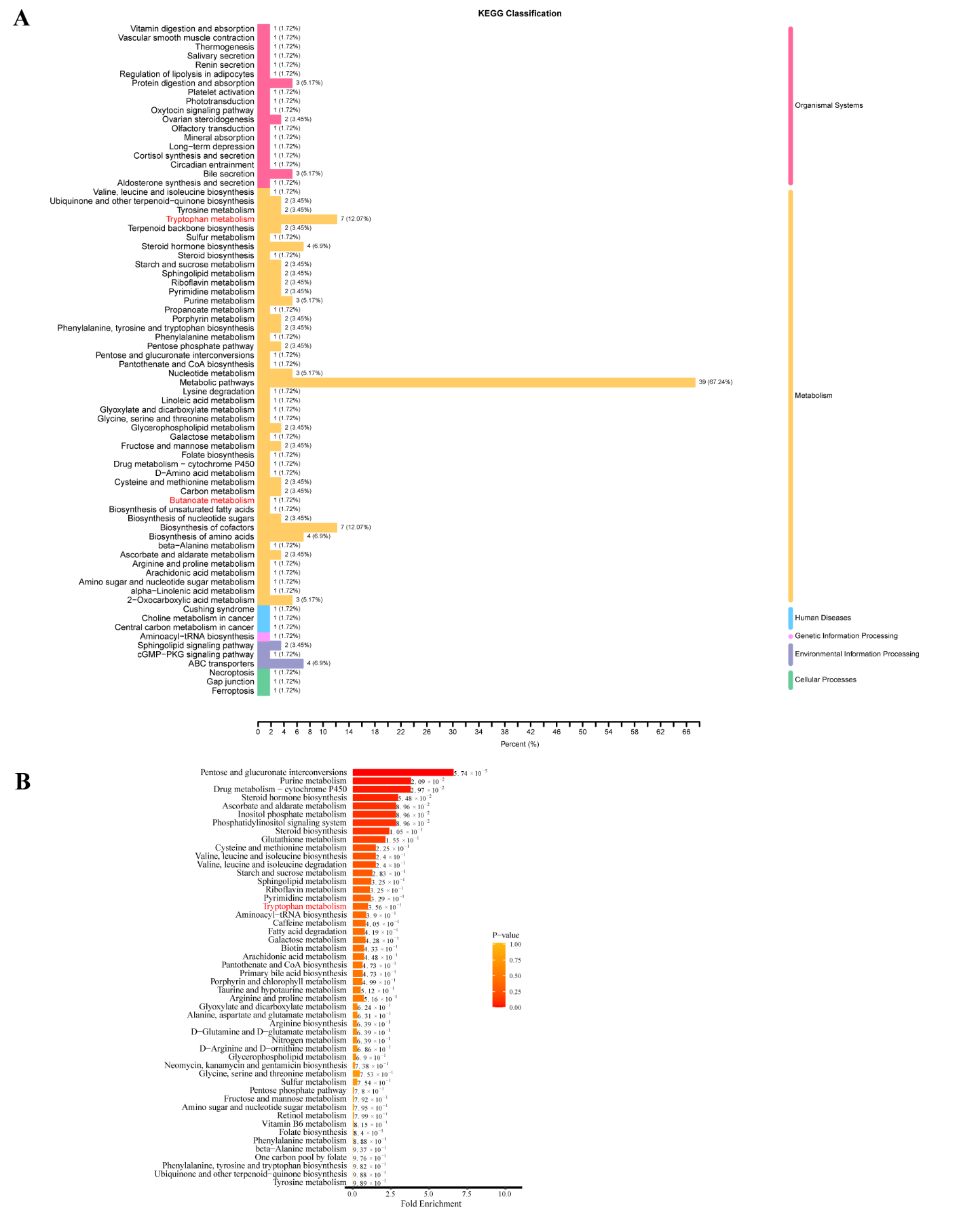


**Supplementary Figure S2. Functional enrichment analysis of plasma metabolomics** **in MRL/*lpr* mice at week 22.** (A) Predicted KEGG functional classification of metagenomic Unigenes. The y-axis represents the names of KEGG metabolic pathways, and the x-axis represents the number of differential metabolites annotated to each pathway, along with the proportion of these metabolites relative to the total annotated metabolites. (B) Metabolite Set Enrichment Analysis (MSEA) plot displaying the top 50 enriched metabolite sets ranked by *P*-value.


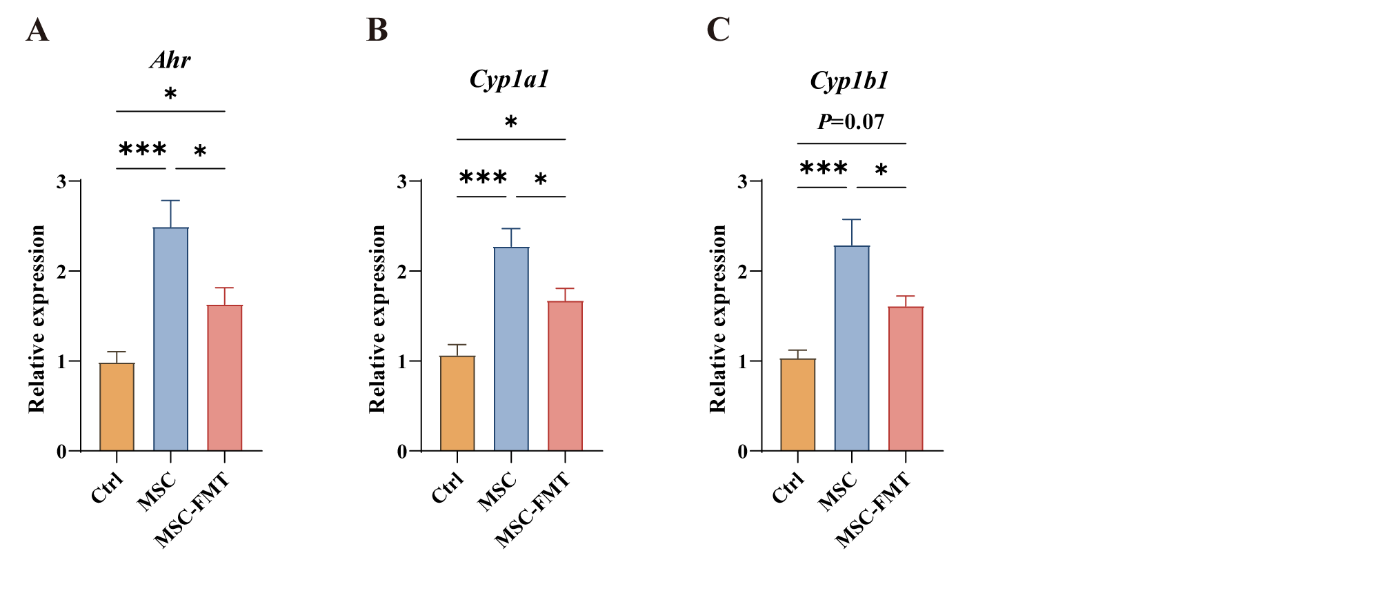
 **Supplementary Figure S3. hUC-MSC transplantation upregulates the expression of colonic Ahr, Cyp1a1, and Cyp1b1 genes in MRL/*lpr* mice at week 22.** (A-C) Quantitative RT-PCR analysis of relative mRNA expression levels of (A) *Ahr*, (B) *Cyp1a1*, and (C) *Cyp1b1* in colonic tissues. N=7 per group. * *P*<0.05, *** *P*<0.001.

**Supplementary Tables**

**Supplementary Table S1**. **Bacterial Species and Their Associated KEGG Metabolic Pathways**

| Species | KEGG Pathway Map | KEGG Orthology | EC Number |
| --- | --- | --- | --- |
| *Lactobacillus johnsonii* | map00380  Tryptophan metabolism | K00626 | 2.3.1.9 |
| *Roseburia* sp. 1XD42_69 | map00380  Tryptophan metabolism | K00626 | 2.3.1.9 |
| *Romboutsia ilealis* | map00380  Tryptophan metabolism | K00382; K07130 | 1.8.1.4; 3.5.1.9 |
| uncultured *Roseburia* sp. | map00380  Tryptophan metabolism | K00626 | 2.3.1.9 |
| *Ligilactobacillus murinus* | map00380  Tryptophan metabolism | K00382 | 1.8.1.4 |
| *Romboutsia ilealis* | map00650  Butanoate metabolism | K00634; K00929; K03737; K20509; K23351; K23352 | 2.3.1.19; 2.7.2.7; 1.2.7.1\|1.2.7.-; 7.2.4.1; -; - |
| *Lactobacillus johnsonii* | map00650  Butanoate metabolism | K00135; K00626; K01575; K01641; K26318 | 1.2.1.16\|1.2.1.79\|1.2.1.20; 2.3.1.9; 4.1.1.5; 2.3.3.10; 1.3.2.4 |
| *Ligilactobacillus murinus* | map00650  Butanoate metabolism | K01575; K01641; K03366; K26318 | 4.1.1.5; 2.3.3.10; 1.1.1.-\|1.1.1.76\|1.1.1.304; 1.3.2.4 |
| *Roseburia* sp. 1XD42_69 | map00650  Butanoate metabolism | K00074; K00248; K00626; K01652; K01653; K01715; K04072; K17865; K18122; K18372; K20509; K23351; K23352 | 1.1.1.157; 1.3.8.1; 2.3.1.9; 2.2.1.6; 2.2.1.6; 4.2.1.17; 1.2.1.10\|1.1.1.1; 4.2.1.55; 2.8.3.-; 3.1.1.114; 7.2.4.1; |

**Supplementary Table S2**. **Antibodies and reagents used in the study.**

| **Antibodies** | **Company** | **Catalog No.** | **Application** | **Dilution fold** |
| --- | --- | --- | --- | --- |
| Purified anti-mouse CD16/32 Antibody | BioLegend | 158002 | FCM | 50 |
| APC-Cy7-conjugated anti-CD19 | BD Biosciences | 552770 | FCM | 80 |
| Percp-Cy5.5-conjugated anti-CD138 | BioLegend | 142510 | FCM | 80 |
| PE-conjugated anti-PD-L2/ CD273 | BD Biosciences | 554689 | FCM | 80 |
| FITC-conjugated anti-IgG | BioLegend | 406001 | FCM | 125 |
| APC-conjugated anti-IgD | BioLegend | 400512 | FCM | 200 |
| BV421-conjugated anti-CD80 antibodies | BD Biosciences | 562612 | FCM | 80 |
| Alexa Fluor 647-conjugated donkey anti-mouse IgG | Invitrogen | A21236 | IF | 400 |
| Alexa Fluor 488-conjugated rat anti-mouse complement 3 (C3) | Novus Biologicals | NB200-540 | IF | 100 |
| CD19 Monoclonal Antibody | eBioscience | 14-0194-82 | IF | 200 |
| Anti-Syndecan-1 antibody (CD138) | Abcam | ab128936 | IF | 50 |
| Rabbit anti-Mouse Tight junction protein ZO-1 antibody | Alpha Diagnostic International | TJP1-A | IF | 250 |
| AhR (D5S6H) Rabbit mAb #83200 | Cell Signaling Technology | 83200 | IF | 100 |
| Donkey anti-Rabbit IgG Secondary Antibody, Alexa Fluor 488 | Invitrogen | A-21206 | IF | 200 |
| Donkey anti-Rabbit IgG Secondary Antibody, Alexa Fluor 594 | Invitrogen | A-21207 | IF | 200 |
| Goat anti-Rat IgG Secondary Antibody, Alexa Fluor 488 | Invitrogen | A-11006 | IF | 200 |

FCM: Flow cytometry. IF: immunofluorescence.

**Supplementary Table S3**. **Primers used for qPCR**

| Gene | Forward primer | Reverse primer |
| --- | --- | --- |
| *ACTB* | CATTCCAAATATGAGATGCGTTGT | GCATTACATAATTTACACGAAAGC |
| *AHR* | ATTGTGCCGAGTCCCATATC | AAGCAGGCGTGCATTAGACT |
| *CYP1A1* | TCAGCTCAGTACCTCAGCCA | CATGGCCCTGGTGGATTCTT |
| *CYP1B1* | GACGCCTTTATCCTCTCTGCG | ACGACCTGATCCAATTCTGCC |
| *Actb* | TGCTTCTAGGCGGACTGTTAC | AACCAACTGCTGTCGCCTT |
| *Ahr* | TGAACACAGAGTTAGACCGCC | TTGCAAATCCTGCCAGTCTCT |
| *Cyp1a1* | GACCCTTACAAGTATTTGGTCGT | GGTATCCAGAGCCAGTAACCT |
| *Cyp1b1* | CACCAGCCTTAGTGCAGACAG | GAGGACCACGGTTTCCGTTG |
